# Hyperphenylalaninemia and serotonin deficiency in *Dnajc12*-deficient mice

**DOI:** 10.1038/s42003-024-07360-6

**Published:** 2024-12-18

**Authors:** Yunqing Cao, Oliver Popp, Niccolo Milani, Fatimunnisa Qadri, Ralf Kühn, Philipp Mertins, Michael Bader, Natalia Alenina

**Affiliations:** 1 Max Delbrück Center for Molecular Medicine in the Helmholtz Association (MDC), Berlin, Germany; 2 Charité - University Medicine, Berlin, Germany; 3 DZHK (German Center for Cardiovascular Research), Partner Site Berlin, Berlin, Germany; 4 Institute for Biology, University of Lübeck, Lübeck, Germany

**Keywords:** Gastrointestinal hormones, Neuroendocrine diseases

## Abstract

Serotonin exerts numerous neurological and physiological actions in the brain and in the periphery. It is generated by two different tryptophan hydroxylase enzymes, TPH1 and TPH2, in the periphery and in the brain, respectively, which are members of the aromatic amino acid hydroxylase (AAAH) family together with phenylalanine hydroxylase (PAH), degrading phenylalanine, and tyrosine hydroxylase (TH), generating dopamine. In this study, we show that the co-chaperone DNAJC12 is downregulated in serotonergic neurons in the brain of mice lacking TPH2 and thereby central serotonin. DNAJC12 has been described to regulate the stability of PAH and mutations in its gene cause hyperphenylalaninemia and neurological symptoms in patients. We show that DNAJC12 also binds and stabilizes TPH1 and TPH2 in transfected cells. In order to clarify the importance of DNAJC12 in the regulation of neurotransmitter synthesis and phenylalanine degradation in vivo, we generated DNAJC12-deficient mice. These mice show reduced levels and activity of PAH, TPH2, and TPH1 in liver, brain, and pineal gland, respectively, and experience hyperphenylalaninemia and central and peripheral serotonin deficiency. These data support a pivotal role of DNAJC12 in the regulation of AAAH and thereby in neurotransmitter synthesis and phenylalanine homeostasis.

## Abstract (web summary)

Characterization of newly generated Dnajc12-deficient mice supports a pivotal role of DNAJC12 in the regulation of aromatic amino acid hydroxylases and, thereby, in the synthesis of neurotransmitters serotonin and dopamine, as well as in phenylalanine homeostasis.

## Introduction

Serotonin (5-hydroxytryptamine, 5-HT) is a major monoamine neurotransmitter that communicates with multiple receptor subtypes (5-HT1-7) throughout the nervous system and in the periphery to regulate key physiological and behavioral functions. Its dysfunction has been implicated in the pathogenesis of psychiatric disorders, most notably depression, anxiety-, and obsessive–compulsive disorders^1^ and a multitude of diseases affecting peripheral organs^2^. The synthesis of 5-HT is initiated by conversion of the essential amino acid tryptophan (Trp) to 5-hydroxytryptophan (5-HTP) by the rate-limiting enzyme tryptophan hydroxylase (TPH), followed by its conversion to 5-HTP by aromatic amino acid decarboxylase (AADC). Two different isoforms of TPH, TPH1 and TPH2, control peripheral and central serotonin systems in vertebrates, respectively^3,4^. Since serotonin does not cross the blood brain barrier, these two systems are totally independent of each other. TPH2 expression and 5-HT synthesis is limited to a small amount of neurons in the hindbrain raphe nuclei. Besides the serotonin-synthesizing machinery, these cells carry proteins responsible for its degradation (monoamine oxidase A, MAOA), packaging into vesicles (vesicular monoamine transporter 2, VMAT2), and reuptake (serotonin transporter, SERT), as well as 5-HT1A autoreceptors which regulate serotonin synthesis and release in a negative feedback loop. The molecular mechanisms involved in this feedback are not fully understood.

TPH1 and TPH2 belong to the family of aromatic amino acid hydroxylases (AAAH) together with tyrosine hydroxylase (TH), responsible for dopamine synthesis, and phenylalanine hydroxylase (PAH) which metabolizes phenylalanine (Phe) to tyrosine^5^. We could recently show that PAH also contributes to peripheral serotonin generation in mammals by efficiently hydroxylating Trp in the liver^6^. Several studies provided evidence that DNAJC12 may regulate the activity of these enzymes by the formation of higher order complexes stabilizing them^7,8^. DNAJC12 belongs to the DNAJ/HSP40 family of heat shock proteins which serve as co-chaperones for HSP70 proteins to assist in the folding of other proteins^9–12^. By yeast-two-hybrid screens an interaction of DNAJC12 with TPH1, TPH2, and TH had already been suggested^13^. Accordingly, Anikster et al. showed that DNAJC12 binds to TPH2, TH and PAH in transfected HEK293 cells^8^. Moreover, patients with mutations in the *DNAJC12* gene exhibit reduced concentrations of 5-hydroxyindole acetic acid (5‐HIAA) and homovanillic acid (HVA), degradation products of serotonin and dopamine, respectively, in the cerebrospinal fluid^7,8,14^ indicating impaired TPH2 and TH activities and decreased serotonin and dopamine synthesis, respectively. Consequently, these patients often present parkinsonism, dystonia and intellectual disablilities and treatment with the products of TPH2 and TH metabolism, 5-HTP and L-3,4-dihydroxyphenylalanine (L-DOPA), can ameliorate their symptoms^15–17^. The most consistent phenotype of patients with *DNAJC12* mutations, however, is hyperphenylalaninemia (HPA) based on a reduced activity of PAH in the liver, which leads to the accumulation of its substate, Phe, in the blood^7,8,14,15^. These increased Phe levels may contribute to the reduced dopamine and serotonin generation in these patients, since Phe has been shown to be a competitive inhibitor of TH and TPH enzymes^18,19^.

Collectively, this body of evidence suggests that DNAJC12 is a key factor influencing 5-HT synthesis through its direct interaction with TPH2. In this study we discovered DNAJC12 in a proteomics screen for proteins which are downregulated in the absence of serotonin in raphe nuclei of TPH2-knockout (KO) mice. We showed its interaction with TPH enzymes in cultured cells. Consequently, we generated *Dnajc12* gene deleted mice and confirmed HPA and central serotonin deficiency in these animals.

## Results

### Identification of DNAJC12 as a potential factor associated with serotonin synthesis regulation through High-throughput Proteomics

**Fig. 1:**
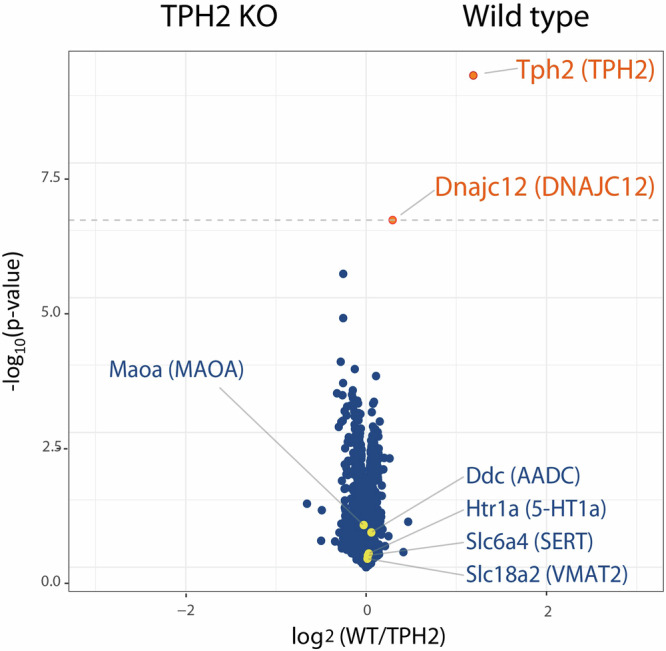
Differentially expressed proteins in Raphe nuclei of TPH2 KO mice. Volcano plot displaying significantly altered proteins (FDR = 0.01, dashed line) identified through proteomics analysis of raphe punches obtained from TPH2 KO and wild type (WT) mice. The labels indicate the proteins’ gene symbols, followed by the protein name in parentheses. Proteins highlighted in yellow denote markers of serotonergic neurons. Proteins highlighted in orange display differential abundance changed between TPH2 and WT samples. The Log2 Fold Change (FC) represents the difference in protein abundance between TPH2 KO and WT, with positive values indicating upregulation in TPH2 KO relative to WT. Proteins downregulated in TPH2 KO compared to WT are positioned on the right side of the plot.

To identify proteins whose expression is affected by serotonin deficiency in the brain, samples enriched in somas of serotonergic neurons, isolated from TPH2 KO mice, lacking brain 5-HT^20^, and wild type C57Bl/6 N (WT) mice, were used for proteomic analysis at the Proteomics Platform of the MDC. The analysis (False Discovery Rate (FDR) = 0.01), revealed 2 differentially expressed proteins, TPH2 and DNAJC12, both downregulated in the TPH2 KO animals (Fig. 1). Marker proteins of serotonergic neurons, such as MAOA, SERT, AADC, VMAT2 and 5-HT1a receptor were also detected in the proteomic analysis, but did not differ between the genotypes. While TPH2 downregulation in TPH2 KO mice was expected, we discovered that DNAJC12 was significantly downregulated in the TPH2 KO animals compared to WT mice.

### DNAJC12 is expressed in serotonergic cells in raphe nuclei

**Fig. 2:**
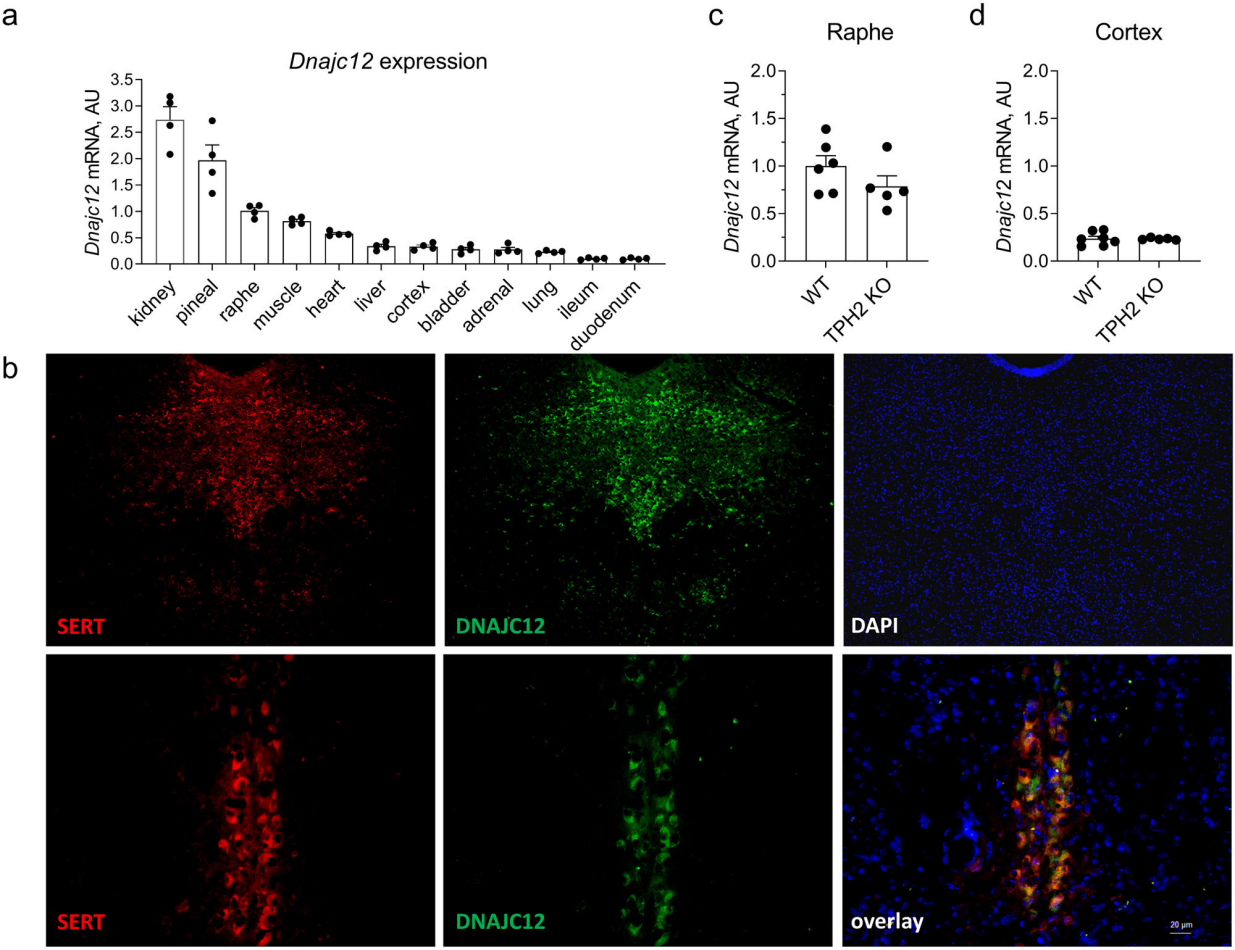
DNAJC12 expression in mouse tissues. **a** Relative DNAJC12 mRNA expression in different organs of wild-type mice (*n* = 4). **b** Immunoflurorescence detection of DNAJC12 protein expression (yellow) in serotonin transporter (SERT)-positive neurons (red) in raphe nuclei of a wild-type mouse. DAPI (blue) was used to stain nuclei. **c, d** Relative DNAJC12 mRNA expression in the raphe nuclei (**c**) and cortex (**d**) of TPH2 KO (*n* = 5) compared to wild type (WT) mice (raphe: *n* = 6, cortex: *n* = 7). Data are presented as mean ± SEM.

To assess the expression levels of *Dnajc12* mRNA across various mouse tissues, quantitative polymerase chain reaction (qPCR) was performed on duodenum, ileum, liver, kidney, bladder, skeletal muscle, heart, lung, adrenal gland, cerebral cortex, raphe nuclei, and pineal gland of WT mice. Remarkably, the highest levels of *Dnajc12* expression were detected in the kidney, pineal gland, and raphe nuclei in the brain (Fig. 2a).

Furthermore, we located DNAJC12 protein expression in mouse brain using immunofluorescence staining. The imaging revealed abundant DNAJC12 in serotonergic neurons of the dorsal raphe nucleus, as indicated by co-localization with the serotonin transporter (SERT), a marker for this cell type (Fig. 2b). Given that the dorsal raphe nucleus serves as a key site for 5-HT synthesis, the specifically high expression of *Dnajc12* mRNA and protein in this area supports its potential involvement in the regulation of serotonin synthesis.

### TPH2-deficiency does not affect Dnajc12 mRNA expression in the brain

To investigate whether the lower DNAJC12 protein levels in raphe nuclei of TPH2 KO mice are due to a decrease in mRNA expression, we conducted qPCR analysis on the raphe nuclei and cortex of WT and TPH2 KO mice. Interestingly, our data revealed no significant change in *Dnajc12* mRNA expression in the brain of TPH2 KO mice (Fig. 2c, d), contrasting with the findings from the proteomic analysis (Fig. 1). This result suggests that the absence of TPH2 mainly affects DNAJC12 expression at the protein level.

### Intracellular interaction between TPH enzymes and DNAJC12

**Fig. 3:**
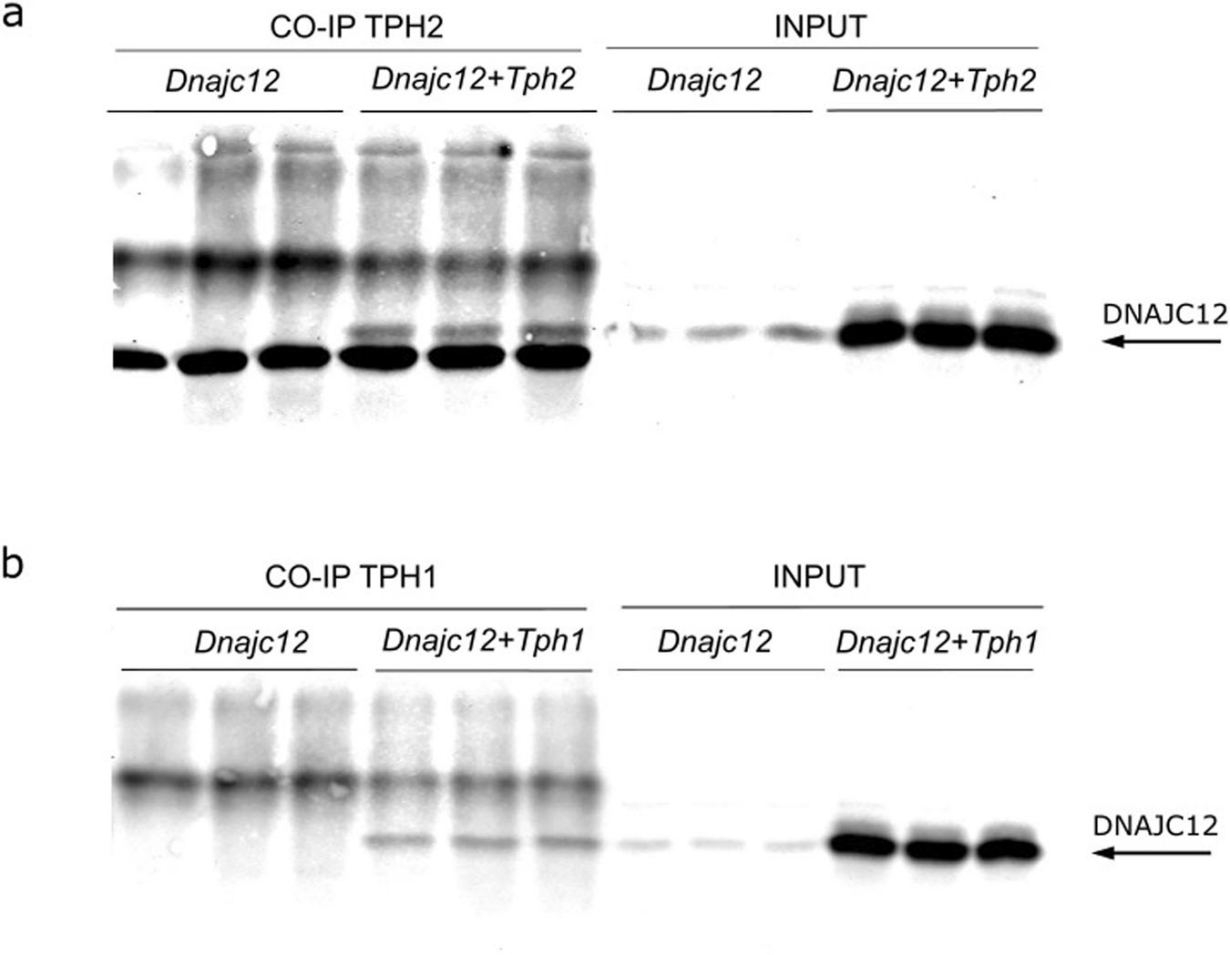
Interaction of DNAJC12 with TPH1 and TPH2. Western Blot with anti DNAJC12 antibodies on proteins co-immunoprecipitated with a TPH1/TPH2 bispecific antibody after transfection of HEK293 cells with expression plasmids for DNAJC12 and TPH2 (**a**) or TPH1 (**b**).

We next tested if DNAJC12 interacts with TPH enzymes, using HEK293 cells transfected with expression plasmids for *Dnajc12* and *Tph1* or *Tph2*. Co-immunoprecipitation experiments were conducted using Protein A/G Agarose coupled with bispecific anti-mouse TPH1/TPH2 antibodies. Subsequent western blot analysis demonstrated that only cells co-expressing TPH1 (Fig. 3a) or TPH2 (Fig. 3b) with DNAJC12 exhibited a discernible signal for precipitated DNAJC12 protein, whereas cells expressing DNAJC12 alone did not yield a positive signal. These results provide supporting evidence for a biologically relevant protein-protein interaction between DNAJC12 and both TPH enzymes.

**Fig. 4:**
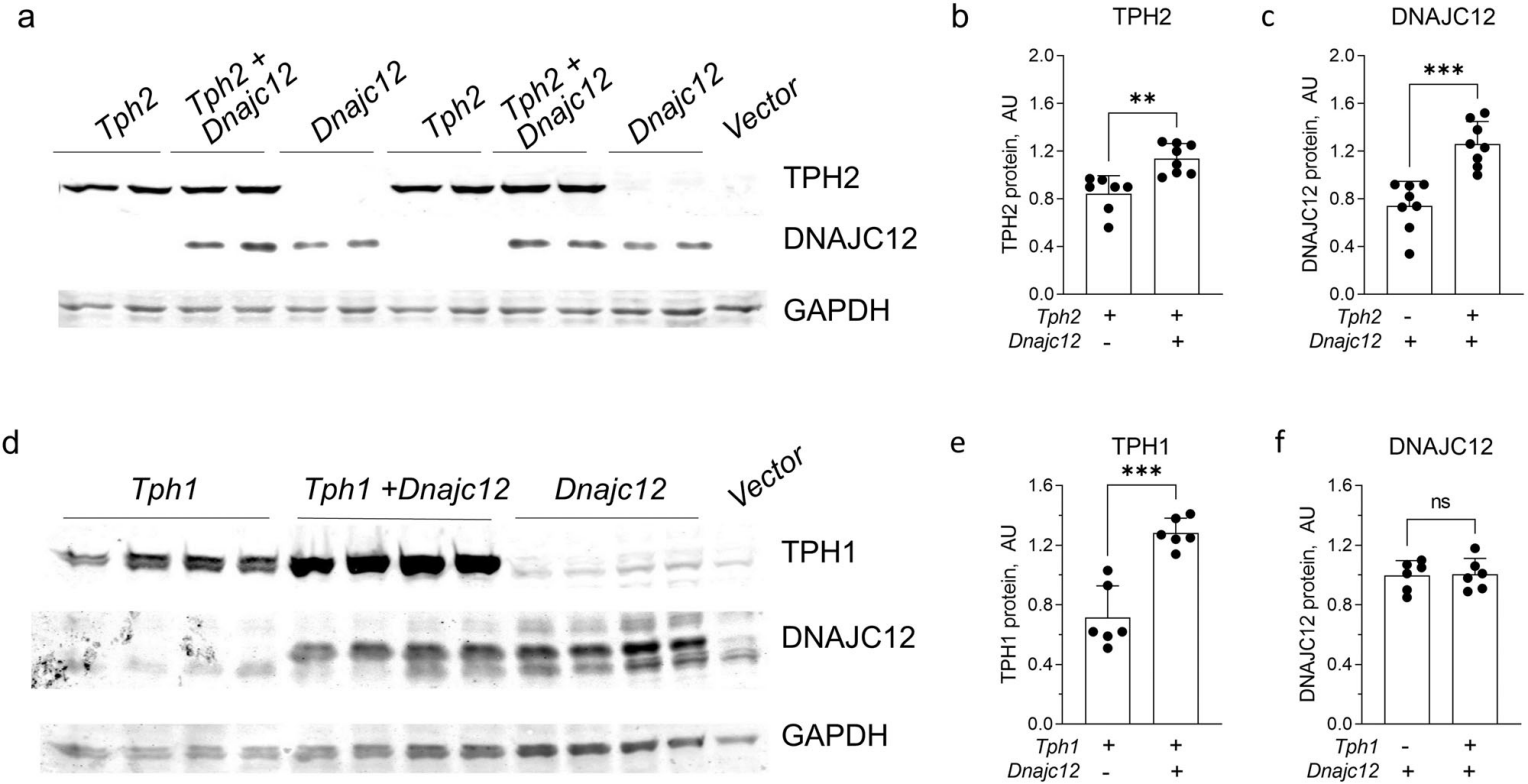
Mutual co-regulation of DNAJC12 and TPH1/TPH2 protein expression. Representative western blot of cells co-transfected with expression plasmids for DNAJC12 and TPH2 (**a**) or TPH1 (**d**). Quantification of TPH2 (**b**) and DNAJC12 (**c**) protein expression in cells transfected with *Tph2* (*n* = 7) or *Dnajc12* (*n* = 8) *or* cotransfected with *Dnajc12* and *Tph2* (*n* = 8). Quantification of TPH1 (**e**) and DNAJC12 (**f**) protein expression in cells transfected with *Tph1 or Dnajc1 or* cotransfected with *Dnajc12* and *Tph1* (*n* = 6). GAPDH was used loading control. **p < 0.01, ***p < 0.001, ns, not significant, Student’s *t*-test. Data are presented as mean ± SEM.

Moreover, a significant enhancement in the TPH protein signals was noted in HEK293 cells co-expressing DNAJC12 and either TPH1 or TPH2 and, vice versa, DNAJC12 protein levels were increased in TPH2 (but not in TPH1) overexpressing cells (Figs. 3 and 4). These findings together with the downregulation of DNAJC12 protein observed in the raphe nuclei of TPH2 KO mice (Fig. 1) implies a positive correlation between DNAJC12 and TPH2, suggesting that their interaction leads to mutual stabilization.

**Fig. 5:**
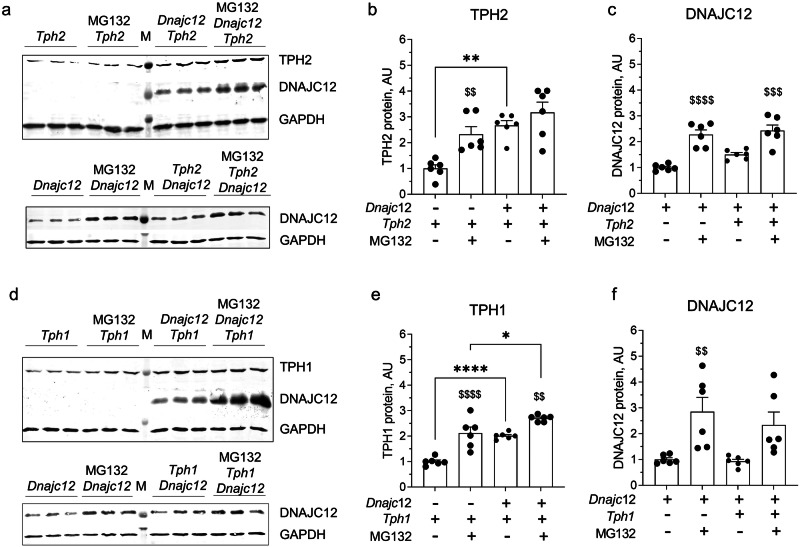
Regulation of proteasomal degradation of TPH1 and TPH2 by DNAJC12. Representative western blot of cells co-transfected with expression plasmids for *Dnajc12* and *Tph2* (**a**) or *Tph1* (**d**), with or without 10 µM MG132 treatment. Quantification of TPH2 (**b**) and TPH1 (**e**) expression in cells cotransfected with *Dnajc1*2 and *Tph2 (B)* or *Tph1* (**e**) and treated with MG132 (*n* = 6). Quantification of DNAJC12 expression in cells cotransfected with *Dnajc12 and Tph2* (**C**) or *Tph1* (**f**) and treated with MG132 (*n* = 6). GAPDH was used loading control. *p < 0.05, **p < 0.01, ****p < 0.0001; ^$$^*p* < 0.01, ^$$$^*p* < 0.001, ^$$$$^*p* < 0.0001 MG132-treated versus untreated for the same condition. One-way ANOVA with Sidak multiple comparison. Data are presented as mean ± SEM.

In order to evaluate whether the ubiquitin/proteasome-dependent degradation of TPH1 and TPH2 is affected by DNAJC12, we treated the HEK293 cells with the proteasome inhibitor MG132 after transfection either with TPH2 or TPH1. This compound increased the concentrations of TPH1 and TPH2 to nearly the same level as co-expression of DNAJC12. When DNAJC12 co-expression and MG132 treatment were combined, the concentrations of TPH1 and TPH2 were not or only slightly further increased compared to MG132 alone (Fig. 5a, b, d, e). These data indicate that DNAJC12 stabilizes TPH enzymes mainly by the inhibition of their ubiquitin/proteasome-dependent degradation. Vice versa, DNAJC12 was stabilized by MG132 and TPH2 co-expression had no additional effect (Fig. 5c). Again, TPH1 co-expression did not alter DNAJC12 levels (Fig. 5f) in HEK293 cells. Thus, TPH2 (but not TPH1) also stabilizes DNAJC12 by inhibition of ubiquitin/proteasome-dependent degradation.

### DNAJC12 genetic deletion results in downregulation of TPH proteins in mice

**Fig. 6:**
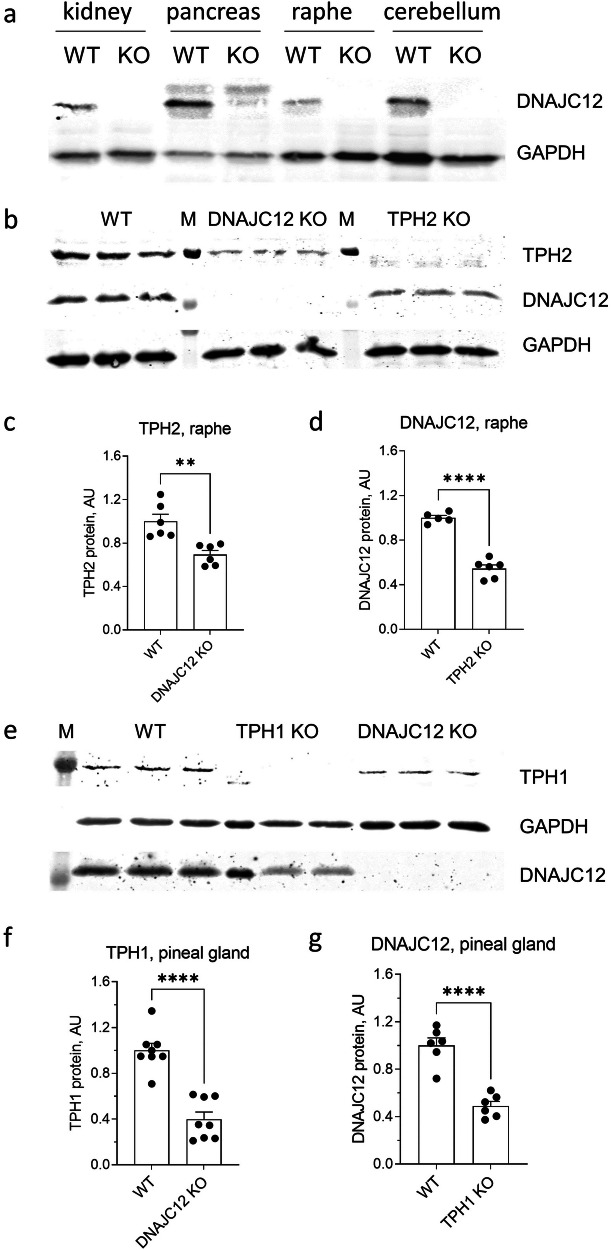
Gene expression in DNAJC12 KO mice. **a** Western blot with anti-DNAJC12 antibodies on kidney, pancreas, raphe nuclei, and cerebellum of wild type (WT) and DNAJC12 KO mice. **b** Representative western blot for DNJAC12 and TPH2 in raphe nuclei of DNAJC12 KO, TPH2 KO and wild type (WT) mice. Quantification of TPH2 expression in the raphe nuclei of DNAJC12 KO (*n* = 6) and WT (*n* = 6) mice (**c**) and of DNAJC12 expression in TPH2 KO (*n* = 6) and WT (*n* = 5) mice (**d**). **e** Representative western blot for DNJAC12 and TPH1 in the pineal gland of DNAJC12 KO, TPH1 KO, and wild type (WT) mice. Quantification of TPH1 expression in the pineal gland of DNAJC12KO (*n* = 8) and WT (*n* = 8) mice (**f**) and of DNAJC12 expression in TPH1 KO (*n* = 6) and WT (*n* = 6) mice (**g**). GAPDH was used as loading control. **p < 0.01, ****p < 0.0001. Student’s *t*-test. Data are presented as mean ± SEM.

To investigate whether the interaction of DNAJC12 with AAAHs is important for their functions in vivo, we utilized CRISPR/Cas9 technology to generate a DNAJC12 KO mouse. We tested DNAJC12 protein expression in organs known to exhibit high DNAJC12 expression, including the kidney, pancreas, raphe nuclei, and cerebellum. Western blot analysis, employing anti-DNAJC12 antibodies, confirmed the absence of DNAJC12 protein in the newly generated animals (Fig. 6a).

We next used this model to quantify the protein levels of TPH enzymes. In the raphe nuclei, the primary site for serotonin synthesis in the brain, western blot revealed a significant downregulation of TPH2 protein expression in the DNAJC12 KO mouse (Fig. 6b, c). Conversely, DNAJC12 protein was also downregulated in TPH2 KO mice (Fig. 6b, d), thus corroborating the proteomics data (Fig. 1). These results support a crucial role of DNAJC12 in maintaining normal TPH2 protein expression in the raphe nuclei. Moreover, the interaction between DNAJC12 and TPH2 is bidirectional also in vivo, as the absence of one protein results in the downregulation of the other.

Next, we tested whether the expression of TPH1 is also affected by the absence of DNAJC12, beyond TPH2 in the raphe nuclei. Western blot analysis disclosed a significant downregulation of TPH1 protein levels in the pineal gland of DNAJC12 KO mice compared to the control group (Fig. 6e, f). DNAJC12 was also downregulated in the pineal glands of TPH1 KO mice (Fig. 6e, g). These findings support a role of DNAJC12 also in the regulation of TPH1.

### DNAJC12-deficiency affects central and peripheral serotonin

**Fig. 7:**
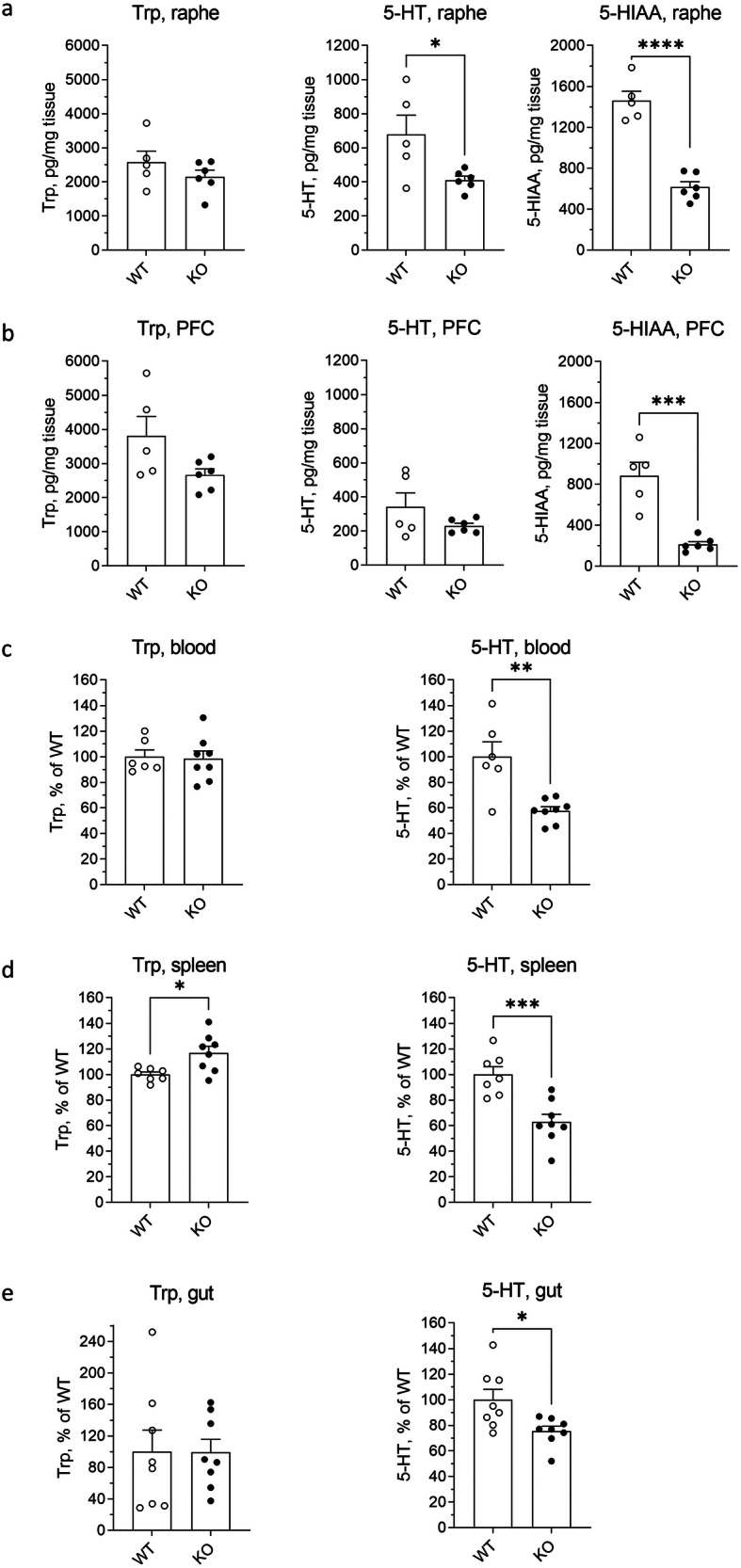
Serotonin metabolism in DNAJC12 KO mice. HPLC measurements of tryptophan (Trp left panel), serotonin (5-HT, middle panel), and 5-hydroxyindoleacetic acid (5-HIAA, right panel in **a** and **b**) in raphe nuclei (WT: *n* = 5, DNAJC12 KO: n = 6) (**a**), prefrontal cortex (WT: *n* = 5, DNAJC12 KO: *n* = 6) (PFC, **b**), blood (WT: *n* = 6, DNAJC12 KO: *n* = 8) (**d**), spleen (WT: *n* = 7, DNAJC12 KO: *n* = 8) (**e**), and gut (WT: *n* = 8, DNAJC12 KO: *n* = 8) (**f**) of DNAJC12 KO (KO) and wild type (WT) mice. *p < 0.05, **p < 0.01, 5, ***p < 0.001, ****p < 0.0001, Student’s *t*-test. Data are presented as mean ± SEM.

**Fig. 8:**
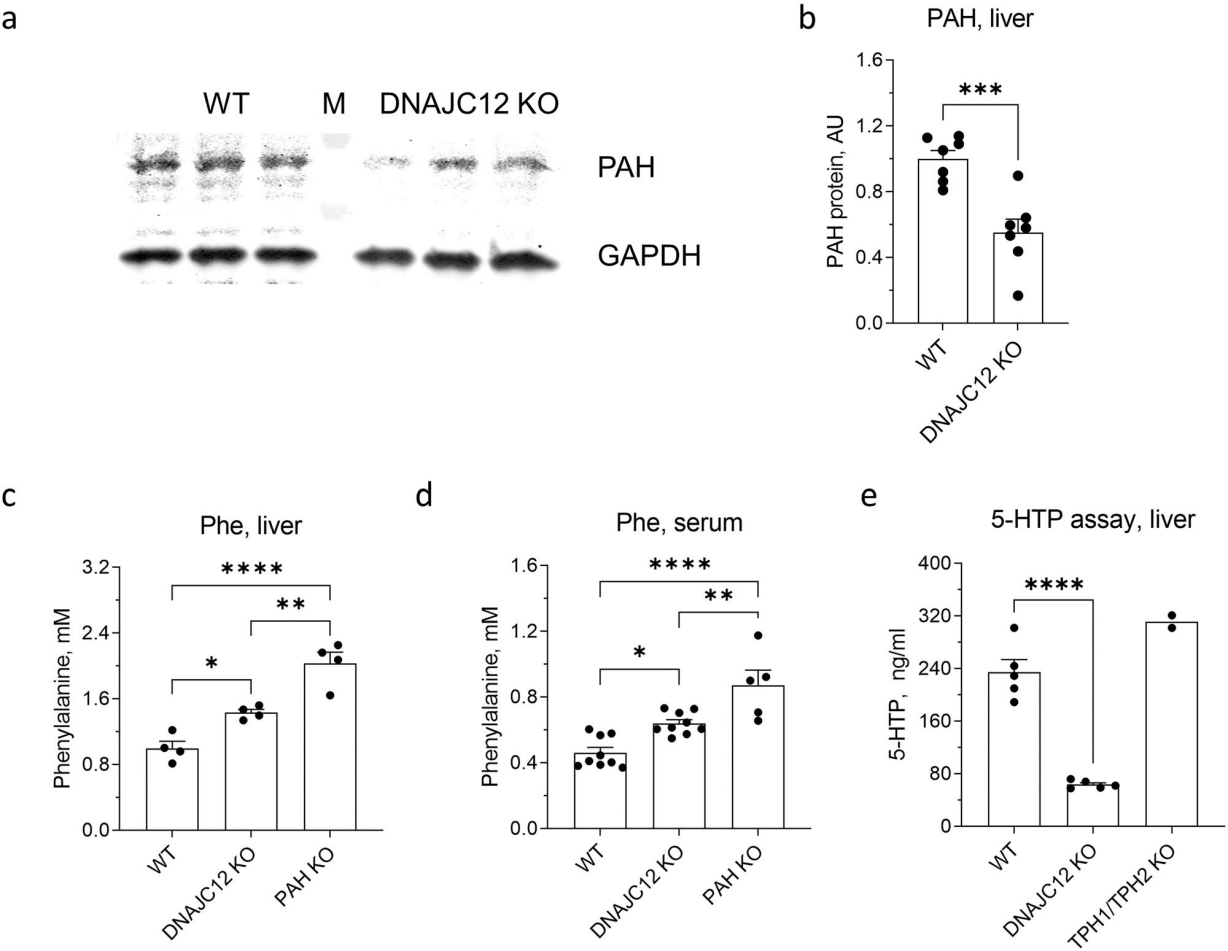
PAH activity in DNAJC12 KO mice. **a** Representative western blot for PAH in the liver of DNAJC12 KO and wild-type (WT) mice. **b** Quantification of PAH expression in the liver of DNAJC12 KO (*n* = 7) mice in comparison to WT (*n* = 7). GAPDH was used as loading control. GAPDH was used as loading control. Phe concentrations in the liver (**c**) and serum (**d**) of DNAJC12 KO and PAH^enu2^ (PAH KO) mice relative to wild-type (WT) mice (*n* = 4). **e** 5-HTP in vitro generation assay in the liver of DNAJC12 KO (*n* = 5), WT (*n* = 5) and TPH1/TPH2-double KO (*n* = 2) mice. *, *p* < 0.05, **p < 0.01, ****p < 0.0001. **b**: Student’s *t*-test; **c–e:** One-way ANOVA wi*t*h Tukey’s multiple comparisons. Data are presented as mean ± SEM.

The reduction in TPH2 protein levels in the brain of DNAJC12 deficient mice has also consequences on serotonin levels in this tissue. Accordingly, while the 5-HT precursor Trp is unchanged, 5-HT is significantly reduced in raphe nuclei of DNAJC12 KO mice, the main site of serotonin synthesis (Fig. 8a). The concentration of the 5-HT metabolite, 5-HIAA, showed a more pronounced reduction not only in the raphe nuclei but also in a serotonergic projection area, the prefrontal cortex (Fig. 7a, b). A drastic decrease in the degradation product, with only a mild or no reduction in serotonin levels, can be considered an indicator of decreased serotonin production. In such cases, the lower serotonin synthesis rates are compensated by its reduced degradation, as demonstrated in mice with partially reduced TPH2 activity^21^. Moreover, the 5-HT levels in the periphery (blood, spleen, and gut) of DNAJC12 KO mice were lower than in wild-type controls (Fig. 7c–e). These findings indicate that DNAJC12 has a pivotal role for central and peripheral serotonin production. Further research may be needed to fully elucidate the specific functions and mechanisms of DNAJC12 in different tissues and under various physiological conditions.

### DNAJC12-deficiency affects PAH function

In addition to the effects on TPH1 and TPH2 expression, DNAJC12-deficiency resulted in downregulation of PAH protein levels in the liver (Fig. 8a, b). Besides its contribution to peripheral serotonin synthesis^6^, the main function of PAH is to metabolize Phe, catalyzing the pivotal step in its complete breakdown into carbon dioxide and water^22^. To assess the functional status of PAH in DNAJC12 KO mice, we measured Phe concentrations in the serum and liver of DNAJC12 KO mice. Phe concentrations in both tissues was significantly higher than in WT mice but lower than in PAH^enu2^ mice, which were used as a control for the absence of PAH activity (Fig. 8c, d). These findings suggest that the absence of DNAJC12 expression reduces the functionality of PAH.

Furthermore, to quantify PAH activity, we conducted 5-HTP in vitro generation assays with liver tissue of DNAJC12 KO mice. In this assay, the activity of Trp-hydroxylating enzymes is estimated by measuring the accumulation of the 5-HT precursor, 5-HTP, under the conditions inhibiting the activity of AADC. In the liver, the two TPH enzymes, TPH1 and TPH2, are not expressed and PAH is the only enzyme hydroxylating Trp^6^ as confirmed by unchanged 5-HTP generation in double KO mice lacking TPH1 and TPH2 (Fig. 8e). In the liver extracts of DNAJC12 KO mice we observed a significant decrease in the accumulation of 5-HTP in comparison to WT mice (Fig. 8e). These findings support the hypothesis that DNAJC12 is essential for normal PAH expression and function in liver, which is the predominant site of PAH expression.

## Discussion

In this study, we could show that DNAJC12 is expressed among other tissues in serotonergic neurons in the brain and interacts with the serotonin-synthesizing enzymes, TPH1 and TPH2, leading to a mutual stabilization of the interaction partners. We also confirm previous reports presenting comparable DNAJC12 effects on another member of the family of AAAHs, PAH^8^. The DNAJC12-mediated stabilization is physiologically relevant since the activity of these enzymes is significantly impaired in DNAJC12-deficient mice leading to HPA and central serotonin deficiency.

An increasing number of publications describe patients with homozygous mutations in DNAJC12 exhibiting HPA, intellectual disabilities and parkinsonism^8,14,16,17,23–27^. These patients exhibit reduced levels of serotonin and dopamine metabolites in the cerebrospinal fluid indicating impaired TPH2 and TH activities in the brain^8,14,16,23,24^. HPA can be treated by Phe-deficient diet and the intellectual disabilities and parkinsonism are ameliorated by treatment with direct precursors for serotonin and dopamine, thereby circumventing the activities of TPH2 and TH, respectively^16,25^.

The postulated mechanism by which a lack of DNAJC12 elicits these symptoms involves a destabilization AAAHs, such as PAH, TPH1, TPH2, and TH^17^. Accordingly, Anikster et al.^8^, have shown that DNAJC12 interacts with PAH and TPH2 in HEK293 cells and that fibroblasts from patients with DNAJC12 mutations exhibit less PAH protein levels and activity. Moreover, Gallego et al.^7^ showed that fibroblasts of patients with DNAJC12 mutations exhibit significantly lower PAH protein levels after transfection with a corresponding expression plasmid and that cotransfection of DNAJC12 and PAH expression plasmids increases the amount of PAH protein and activity in COS-7 cells. Jung-KC et al.^28^ confirmed the interaction of PAH with DNAJC12 in COS-7 cells and showed that the expression of transfected PAH increases endogenously expressed DNAJC12 protein in these cells. Additionally, they showed that DNAJC12 protein (but not mRNA) levels are reduced in the liver of PAH^enu1^ mice lacking PAH activity. Martinez-Pissaro et al.^29^ recently confirmed that expression of a destabilizing PAH mutation reduces DNAJC12 levels in HepG2 cells and transgenic mouse livers. Employing our novel DNAJC12 KO mice, we confirm these data and additionally show that DNAJC12 is also essential for the stabilization and full activity of PAH and TPH2 in vivo in liver and brain of mice, respectively. Moreover, we provide evidence that also TPH1 is stabilized by DNAJC12 in cell culture and in pineal glands of mice. On the other hand, we provide novel evidence that TPH2 is needed to stabilize DNAJC12 in cell culture and in tissues of mice, as has been shown for PAH^28,29^. Surprisingly, while TPH1 interacts with DNAJC12, it fails to stabilize it in the cell culture and in vivo. Further research is required to explore the mechanisms behind this stabilization and why it is a property exclusive to only one of the TPH enzymes.

How does DNAJC12 increase the protein concentration and activity of AAAHs? DNAJC12 is a co-chaperone of the DNAJ/HSP40 family. After binding to client proteins, in this case, PAH, TPH1, TPH2 and TH, DNAJ proteins recruit HSP70 via their J domains in an ATP-dependent manner^11,12^. They boost the ATPase activity of HSP70 and get released from the complex after ATP cleavage. HSP70 remains bound to the client protein and prevents its degradation and aggregation until ATP is reconstituted by corresponding nucleotide exchange factors. By binding to only a subset of targets, DNAJ proteins induce specificity to the HSP70 chaperoning activity. There are around 50 DNAJ proteins in humans, each with a different target specificity. DNAJC12 seems to be mainly selective for AAAHs, but may have also other interaction partners^9^. For PAH it has been shown that DNAJC12 and HSP70 stabilizes the enzyme by inhibiting ubiquitylation and degradation of the enzyme by the proteasome^28^ which is normally controlling its half-life^30,31^. The ubiquitin-proteasome pathway is also a major determinant of TPH1 stability^32^. Accordingly, we could show that inhibition of the proteasome by MG132 partially mimicked the stabilizing effect of DNAJC12 on TPH1 and TPH2 in transfected cells. Thus, we posit that DNAJC12 may exert its effect on TPH enzymes by preventing their ubiquitylation.

Already several decades ago, it has been shown that TH and TPH enzymes can be bound and activated by 14-3-3 proteins^5,33^. This is a family of highly conserved small chaperone proteins mainly expressed in the brain and exerting a multitude of functions^34^. Off note, the genetic name for this gene family, YWHA, is derived from their role in the activation of TH, TPH1, and TPH2 (tyrosine (Y) and tryptophan (W) hydroxylase (H) activators (A)), since this was the first function described for them^33^. They bind to TH, TPH1 and TPH2 after these enzymes have been phosphorylated at certain sites resulting in their stabilization and enzymatic activation^35–38^. Interestingly, despite that PAH is also regulated by phosphorylation^5,39^ an interaction of this enzyme with 14-3-3 proteins has not yet been described, but this may be due to the predominant expression of these chaperones in the brain of mammals, where PAH is not present.

It remains unclear whether the two chaperones, DNAJC12 and 14-3-3 proteins, independently regulate the levels and activities of AAAHs or whether they interact. It has been shown that another member of the DNAJ family, DNAJC5, can be itself phosphorylated and then also binds a 14-3-3 protein^40^, but there is also a report from Drosophila on coordinated activities of the two chaperone systems^41^. Future studies will be necessary to understand the exact mechanisms by which DNAJC12 and 14-3-3 proteins stabilize and activate AAAHs.

Most likely, DNAJC12 is not involved in the negative feedback loop regulating serotonin synthesis in the raphe nuclei, since it, and consequently also TPH2, is downregulated when serotonin is absent, while one would expect TPH2 upregulation for compensation. Nevertheless, DNAJC12 regulates the serotonin synthesis in the periphery and in the brain and is therefore one of the few common components of these otherwise completely independent systems^3^. It could be exploited pharmacologically to modulate both systems in the same direction, however, in most cases this would not be intended. While an increase in serotonin by TPH2 stimulation is a pharmacological target in the brain, in the periphery a decrease by TPH1 inhibition would be purposed^1,2^.

Taken together, by generating mice lacking DNAJC12, we have shown that this protein is essential in the regulation of the AAAHs, TPH1, TPH2, and PAH, and thereby for neurotransmitter synthesis and phenylalanine homeostasis. This animal model will be useful to study the mechanisms causing HPA, parkinsonism and intellectual disabilities and to test novel therapeutic options in patients with DNAJC12 mutations.

## Materials and Methods

### Animals

Mice were maintained at the animal facility of the Max Delbrück Center, in pathogen-free conditions, in individually ventilated cages, in accordance with the German Animal Protection Law and approved by the Landesamt für Gesundheit and Soziales (Berlin, Germany). We have complied with all relevant ethical regulations for animal use. TPH1 KO^4^ and TPH2 KO^20^ mice were on the C57Bl6/N background. PAH^enu2^ mice (PAH-KO)^42^ were obtained from Jackson Laboratories, USA (#002232). *Dnajc12* mutated mice were generated at the transgenic core facility of the MDC in the course of this study. Adult (12 to 20-week-old) mice of both sexes were used to collect organs for the molecular biological and HPLC analysis.

### Generation of Dnajc12-gene mutated mice

For the generation of *Dnajc12* gene mutated mice two gRNAs with target sites flanking exon 3 (IDT-DNA) were injected together with Cas9 protein (IDT-DNA) and a 711 nt long single-stranded DNA donor template, containing homology regions and two loxP sequences (Genewiz) into pronuclei of C57Bl/6 N mouse zygotes as described^43^. The manipulated zygotes were transferred into pseudo-pregnant CD1 females to obtain mutant founder pups. The genomic DNA of the offspring was analyzed by PCR using primers flanking exon 3 and subsequent sequencing of the PCR fragments. Out of 16 pups, that were born, 2 animals had correct integration of the loxP-containing fragments and 4 animals carried deletions between the two gRNA binding sites, leading to the complete loss of the exon 3. These founders were bred to homozygosity and the resulting DNAJC12-deficient offspring of one strain (DNAJC12-S2272) was used for this study. After the line was established, mice were routinely genotyped using primers G_DNAJC12 fw: 5’-TCA AAC TCA GGA CGT CAG GC and G_DNAJC12 rev1: 5‘-CCA CTG AGC CAT CTG ACC AG using a Taq Polymerase PCR system (NEB) according to the protocol of the manufacturer resulting in generation of 540 and 247 bp long fragments for the wild type (WT) and KO alleles, respectively.

### Proteomics

To collect the samples for proteomics, brains from three TPH2 KO and three C57Bl/6 N control mice, aged 10-12 weeks, were sliced into 300 µm thin sections using a cryotome (Epredia™ CryoStar™ NX70 Kryostat, Thermo Fisher Scientific). Six punches (1 mm) containing the dorsal raphe nuclei from the same mouse were cut out and pooled. To prepare proteins, tissues were incubated for 15 min on ice in 400 μl chilled lysis buffer (8 M Urea, 75 mM NaCl, 50 mM Tris pH 8.0, 1 mM EDTA, 2 μg/ml Aprotinin, 10 μg/ml Leupeptin, 1 mM PMSF, 10 mM NaF, 1:100 Phosphatase inhibitor cocktail 2 (Sigma P5726), 1:100 Phosphatase inhibitor cocktail 3 (Sigma P0044), 5 mM CAA). After centrifugation (10 min, 4 °C, 20,000 g), the protein concentration of the supernatant was estimated with a BCA assay. Subsequently, disulfide bonds were reduced using 5 mM dithiothreitol and alkylated with 10 mM iodoacetamide. This was succeeded by a dilution of 1:4 with 50 mM Tris pH 8.0. For protein digest, LysC (Wako) digestion was conducted at room temperature for 2 hours, maintaining a protein-to-enzyme ratio of 50:1. Following this, an overnight digestion was performed with sequence-grade trypsin (Promega) at the same ratio. Peptides were subsequently acidified and desalted using SepPak desalting columns. For tandem mass tag (TMT; Thermo Fisher Scientific) based proteomic analysis, the methodology outlined in Mertins et al., and Ng et al.^44,45^ was followed closely. In this study, 20 µg of peptides from each sample were labelled using randomly assigned TMT11 (Thermo Fisher Scientific) channels, with channel 9 deliberately left empty. Channel 11 was loaded with a comprehensive bulk sample, comprising a mixture totaling 144 µg from all individual samples. The pooled TMT-plex was then fractionated into 24 fractions using an UltiMate 3000 Systems (Thermo Fisher Scientific). Raw data was acquired on a Q Exactive HF-X orbitrap mass spectrometer (Thermo Fisher Scientific) connected to an EASY-nLC 1200 system (Thermo Fisher Scientific).

For analysis, MaxQuant version 1.6.10.43^46^ was used employing MS2-based reporter ion quantitation. Carbamidomethylation was set as a fixed modification and deamidation of asparagine and glutamine as well as oxidation on methionine were employed as variable modifications. A PIF filter was applied with a threshold of 0.5. For database search, a Uniprot mouse database (2018-07) including isoforms plus a database of common contaminants were used. For protein quantitation only protein groups identified by at least two peptides and by a minimum of one unique peptide were considered for quantitation. Corrected log2-transformed reporter ion intensities were used for quantitation. Protein intensities were filtered for 100% valid values across all samples and normalized by median-MAD before applying a two-sample moderated t-tests between experimental groups^47^. P-values were adjusted using the Benjamini-Hochberg procedure. The mass spectrometry proteomics data and search results have been deposited to the ProteomeXchange Consortium via the PRIDE partner repository^48^. Data are available via ProteomeXchange with the identifier PXD055579.

### Cell culture experiments

HEK293 cells were grown in Dulbecco Modified Eagle Medium-low glucose with pyruvate (Gibco, Paisley, Scotland, UK), supplemented with 10% FBS (Gibco, Paisley, Scotland, UK) and 1% Penicillin/Streptomycin (Sigma, Steinheim, Germany) in a 37 °C humidified 5% CO_2_ incubator.

Expression plasmids for TPH1 (TPH1_OHu22236C_pcDNA3.1( + )), TPH2 (TPH2_OHu18561C_pcDNA3.1(+)) and DNAJC12 (DNAJC12_OHu13037C_pcDNA3.1(+)) were purchased from GenScript Biotech, USA. Plasmids were purified by NucleoBond Xtra Maxi Kit for Plasmid DNA (MACHEREY-NAGEL, REF 740414.50). TPH1, TPH2 and DNAJC12 overexpression in HEK293 cells was achieved by DNA transfection using Lipofectamine™ 3000 Transfection Reagent (Thermo Fisher, Cat. #L3000001) with the empty pcDNA3.1(+) vector as control. The plasmid DNAs were used at a final concentration of 1.25 µg/ml. In some experiments, 10 µM of the proteasome inhibitor MG132 (Sigma M8699) was added to the culture medium 24 hours after transfection. Cells were harvested 48 hours after transfection.

### Quantitative real-time PCR

RNA was isolated from 50–100 mg of snap-frozen tissue using the Trizol method. Tissue was homogenized in 1 ml of Trizol (Invitrogen) using a homogenization system (FastPrep; MPI) according to the protocol of the manufacturer. To remove traces of genomic DNA, samples were treated with recombinant DNase I (Sigma). Nucleic acid concentrations were quantified using NanoDrop (PEQLAB Biotechnologie). cDNA was produced from 1 μg of DNaseI-treated RNA using Moloney Murine Leukemia Virus (M-MLV) reverse transcriptase (Promega Corporation) following manufacturer’s instructions. Real-time PCR was performed with SYBR green master mix reagents (Thermo Fisher Scientific) using the QuantStudio™ 5 Real-Time PCR System (Thermo Fisher Scientific). All primers were purchased from BioTeZ Berlin-Buch. 18S and TBP were used as endogenous reference genes (Table 1). Gene expression was analyzed using the 2^−ΔΔCT^-method^49^.

**Table 1:** Primer sequences

Gene	Forward primer	Reverse primer
Mouse/ human TBP	5′-CCCTATCACTCCTGCCACACC-3′	5′-CGAAGTGCAATGGTCTTTAGGTC-3′
Mouse TPH2	5′-CCTTTGCAAGCAAGAAGGTC-3′	5’-TTGGAAGGTGGTGATTAGGC-3′
Mouse DNAJC12	5′-CTCGCTGGTTTGGAAAGTCCT-3′	5’-ATCGCGTCCATTTACAGCGG-3′
Human TPH2	5′- CCTTTGCAAGCAAGAAGGTC-3′	5’-TTGGAAGGTGGTGATTAGGC-3′
Human DNAJC12	5′-GCACTGGGTTGTCAGAGGTAA-3′	5’-TGGGTTCTTTCTGCTCCGTT-3′

### Immunofluorescence staining of free-floating brain sections

Mice were sacrificed by cervical dislocation. Brains were removed, postfixed in 4% formalin overnight, transferred into 30% sucrose solution and kept at 4 °C until they sink to the bottom of the tubes. The brains were sliced to 40 µm free-floating sections and kept in cold 1x PBS. For immunostaining, sections were transferred into 24 well plates, rinsed 3x with 1x PBS, followed by permeabilization at room temperature (RT) with 0.1% Triton X-40 in 1x PBS for 30 min. Unspecific binding was blocked by incubating the sections in 1% bovine serum albumin for 1 h at RT. Thereafter the incubation solution was discarded and the sections were incubated with primary anti-SERT or anti-DNAJC12 (Abcam, ab167425) antibodies (1:200 and 1:250, respectively) overnight at 4 °C. Next day the sections were rinsed 3x with 1x PBS and incubated with fluorochrome-conjugated secondary antibody (mouse-Anti-Goat-Cy3, Jackson, 1:300 or goat- Anti-Rabbit-Alexa 647, 1:300, Invitrogen) diluted in 1x PBS and incubated for 2 h at RT in the dark. The sections were rinsed 3x in 1x PBS, mounted on to slides, cover-slipped with an anti-fade mounting medium containing DAPI (Fluoromount-G, Thermo Fisher, Germany), and stored at 4 °C protecting from light. Images were taken using a fluorescence-bright field microscope (Keyence BZ-X800, Japan).

### Western blotting

Cells and mouse tissues were lysed in RIPA lysis buffer [20 mM Tris-HCl (pH 7.5), 150 mM NaCl, 1 mM Na2EDTA, 1 mM EGTA, 1% NP-40, 1% sodium deoxycholate and 1 mM AEBSF]. Proteins were separated by 10% SDS-PAGE electrophoresis and transferred to nitrocellulose membranes (Thermo Scientific, USA) in a wet transfer device (Bio-Rad, Hercules, CA, USA). The proteins of interest were detected with primary antibodies listed in Table 2. IRDye® Donkey anti-Mouse, anti-Rabbit and anti-Goat IgG were used where appropriate as secondary antibodies (Table 2). Chemiluminescence was detected with an Odyssey® DLx Imaging System (LI-COR Biosciences, Lincoln, NE, USA). Quantification analysis was done using the ImageJ software.

**Table 2:** Antibodies

Primary antibody	dilution	Host	Company	CatNr
DNAJC12	1:1000	Rabbit	Abcam	ab167425
Tryptophan Hydroxylase	1:1000	Mouse	Sigma	T0678-WH3
TPH1 (D10C10)	1:1000	Rabbit	ThermoFisher	mAb#12339
TPH2	1:1000	Rabbit	ThermoFisher	PA1-778
GAPDH (14C10)	1:1000	Rabbit	Cell Signaling	2118
PAH	1:1000	Goat	Santa Cruz	SC15112
**Secondary antibody**				
IRDye 800CW DαM	1:2000		LI-COR IRDye	926-32212
IRDye 800CW DαG	1:2000		LI-COR IRDye	926-32214
IRDye 800CW DαR	1:2000		LI-COR IRDye	926-32213
IRDye 680CW DαM	1:2000		LI-COR IRDye	926-32222
IRDye 680CW DαG	1:2000		LI-COR IRDye	926-32224
IRDye 680CW DαR	1:2000		LI-COR IRDye	926-32223
IRDye 680CW DαR	1:2000		LI-COR IRDye	926-32223

### Co-immunoprecipitation

The immunoprecipitation process involved the binding of Protein A/G PLUS-Agarose (Santa Cruz, cat. sc-2003) to Anti-DNAJC12 antibody (Abcam, ab167425), through an incubation at 4 °C overnight on a rotator. Lysis of the cells overexpressing TPH1, TPH2, DNAJC12, TPH1 + DNAJC12, and TPH2 + DNAJC12 was performed using Lysis Buffer consisting of 150 mM NaCl, 50 mM Tris pH 7.5, 1% IGPAL-CA-630 (Sigma I8896), 5% Glycerol, and MS-SAFE Protease and Phosphatase Inhibitor (Sigma MSSAFE-5VL). 1 mg of cell lysate per sample was added to the beads, followed by an overnight incubation at 4 °C on a rotator. After centrifugation at 4 °C for 10 min at 14,000 g, the beads were washed 2 times with Wash Buffer (150 mM NaCl, 50 mM Tris pH 7.5, 5% Glycerol, 0.05% IGPAL), and 2 times with PBS. Protein was released from the beads by heating the samples at 95 °C for 5 minutes with ROTI®Load 1 buffer (Roth K929.1). The presence of TPH1 and TPH2 digested from the beads was assessed through Western blot analysis.

### Phenylalanine assay

Serum and liver samples from WT, PAH^enu2^, and DNAJC12 KO mice were diluted 1/40 and analyzed by the Phenylalanine Assay Kit (Sigma MAK005) according to manufacturer’s instructions. The fluorescence (λex = 535/λem = 587 nm) was measured in a Infinite® 200 PRO multimode plate reader (Tecan, Switzerland).

### HPLC analysis

For the quantification of monoamines in mouse organs, frozen tissues were homogenized in lysis buffer containing 1.68% PCA (Perchloric acid, Sigma 311421), 568 μM AA (ascorbic acid, Sigma A7506) and 2 mg/mL sodium metabisulfite. After centrifugation (20 min at 4 °C, 15-20000 g) supernatants were transferred to Eppendorf tubes and stored at −80 °C until use.

Trp, 5-HTP, 5-HT, and 5-HIAA were quantified using high-sensitive reversed-phase high-performance liquid chromatography (HPLC) with fluorometric detection. Samples were injected into the chromatographic system (SIL-20A/AC, 10 μL loop, Shimadzu, Kyoto, Japan) and separated over a C18 reversed-phase column (LipoMare C18, AppliChrom, Oranienburg, and ProntoSIL 120 C18 SH, VDS Optilab, Berlin) at 20 °C in a 10 mM potassium phosphate buffer (pH 5.0) (Sigma, Steinheim, Germany) with 5% methanol (Roth, Karlsruhe, Germany) and a flow rate of 1 mL/min. The excitation wavelength was 295 nm and the fluorescent signal was measured at 345 nm. The CLASS-VP software (RF-20Axs System, Shimadzu, Kyoto, Japan) was used to analyze the peak parameters of chromatographic spectra. The calculation of substance levels was based on external standard values. Amounts of 5-HT, 5-HIAA, and Trp were normalized to the wet tissue weight.

### In vitro 5-HTP generation assay

Frozen mouse organs were lysed in a 75 mM Tris-Acetate buffer, pH 7.5. The lysates were mixed with a pre-incubation buffer, containing 2 mg/ml Catalase, 25 mM DTT, and 100 μM Fe(NH4)2(SO4)2 (FAS), and incubated for 10 minutes at 30 °C. The 5-HTP synthesis reaction was run for 30 minutes at 37 °C after the addition of an assay buffer composed of 1 mM Trp, 250 μM 6MPH4, 2 mM NSD1015, and 15 mM Tris-Acetate at pH 6.4. The reaction was subsequently stopped by adding 12 μL PCA. The 5-HTP levels were assessed by HPLC.

### Statistics and reproducibility

Statistical significances of observed differences were evaluated by two-tailed *t*-tests and one-way ANOVA with Sidak’s or Tukey’s multiple comparison, when appropriate, using Prism 7 software (GraphPad). *p* values < 0.05 were considered significant. Results are expressed as means ± s.e.m. Each dot in the bar graphs represents one independent experiment or tissue sample (at least 4).

### Reporting summary

Further information on research design is available in the Nature Portfolio Reporting Summary linked to this article.

## Supplementary information

Supplementary Information

Description of Additional Supplementary File

Supplementary data 1

Reporting summary

## Data Availability

All data generated or analyzed during this study are included in this published article. The proteomics data are available via the ProteomeXchange Consortium with identifier PXD055579. The source data behind the graphs in the paper and the original Western blots can be found in Supplementary Data 1 and Supplementary Figs. files, respectively. All other data are available from the corresponding author on request.
