# Supplementary material for: Hyperphenylalaninemia and serotonin deficiency in Dnajc12-deficient mice
Source: Commun Biol. 2024 Dec 18;7:1641. doi: 10.1038/s42003-024-07360-6 (PMC11655531; doi:10.1038/s42003-024-07360-6)
Supplement: Supplementary file 1 — Supplementary Information [file 42003_2024_7360_MOESM1_ESM.pdf]

Figure 3a

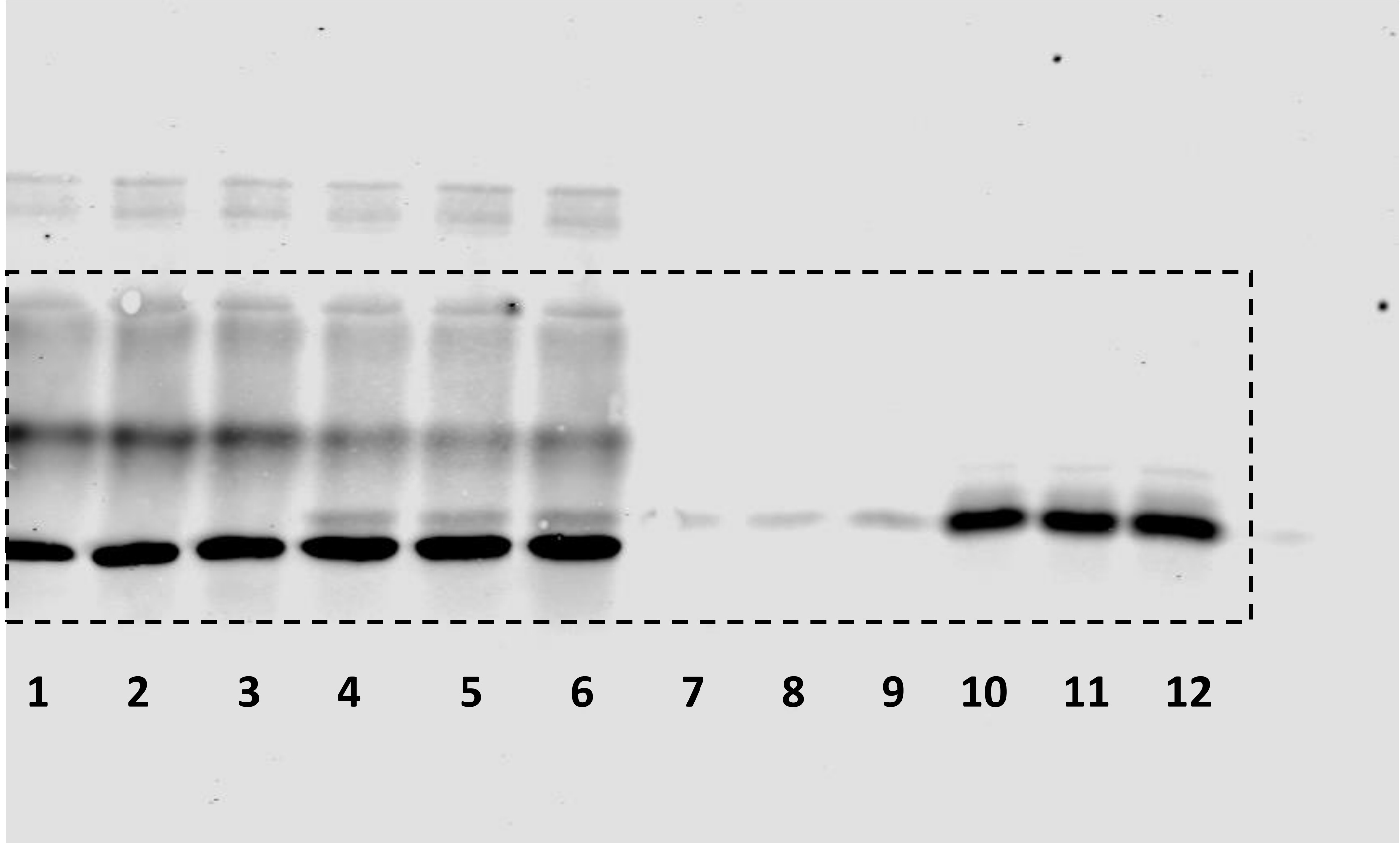

anti-DNAJC12

1-3: *Dnajc12* IP-TPH2
4-6: *Dnajc12+Tph2* IP-TPH2
7-9: *Dnajc12* input
10-12: *Dnajc12+Tph2* input

Figure 3b

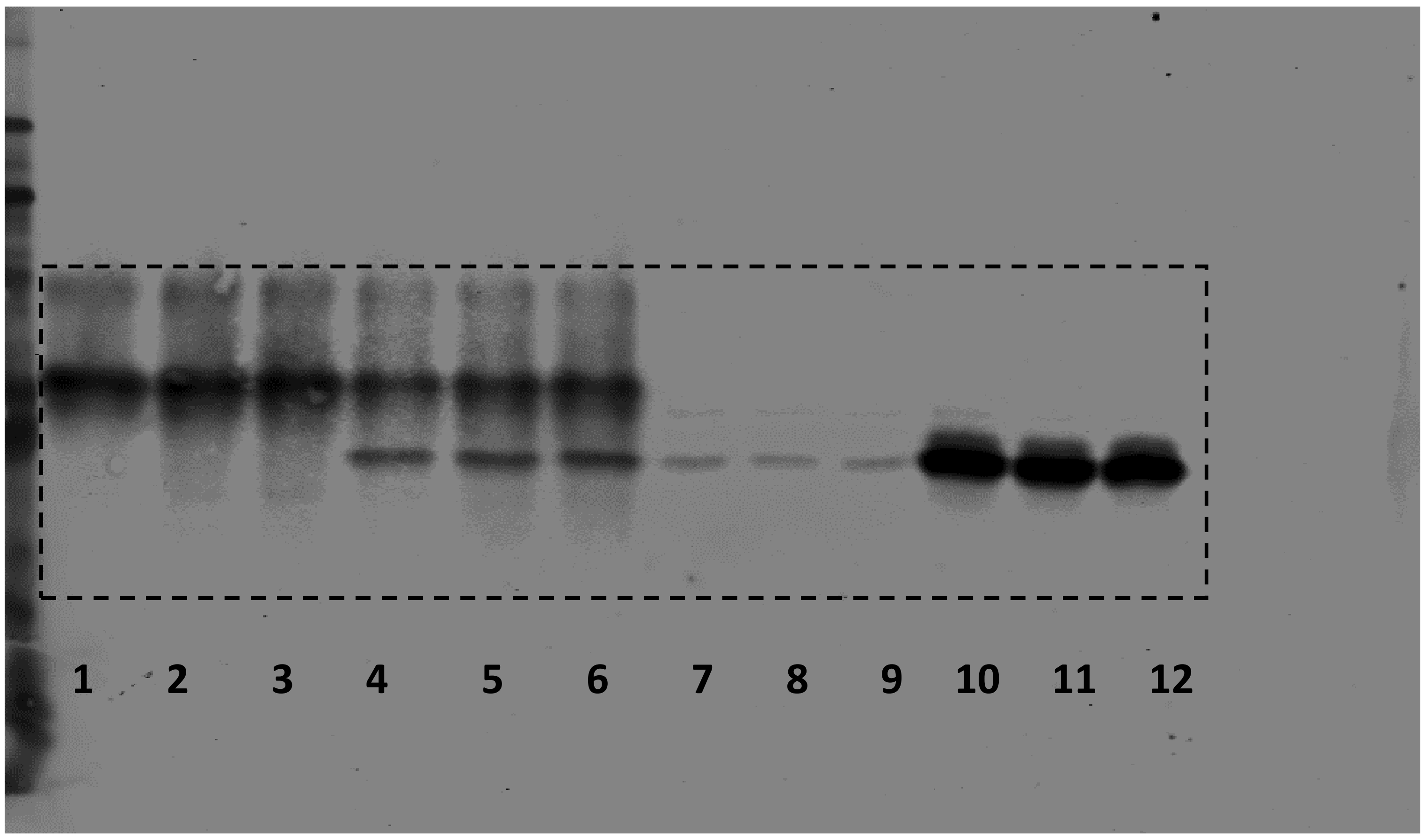

anti DNAJC12

1-3: Dnajc12 IP-TPH1
4-6:Dnajc12+Tph1 IP-TPH1
7-9: Dnajc12 input
10-12: Dnajc12+Tph1 input

Figure 4a

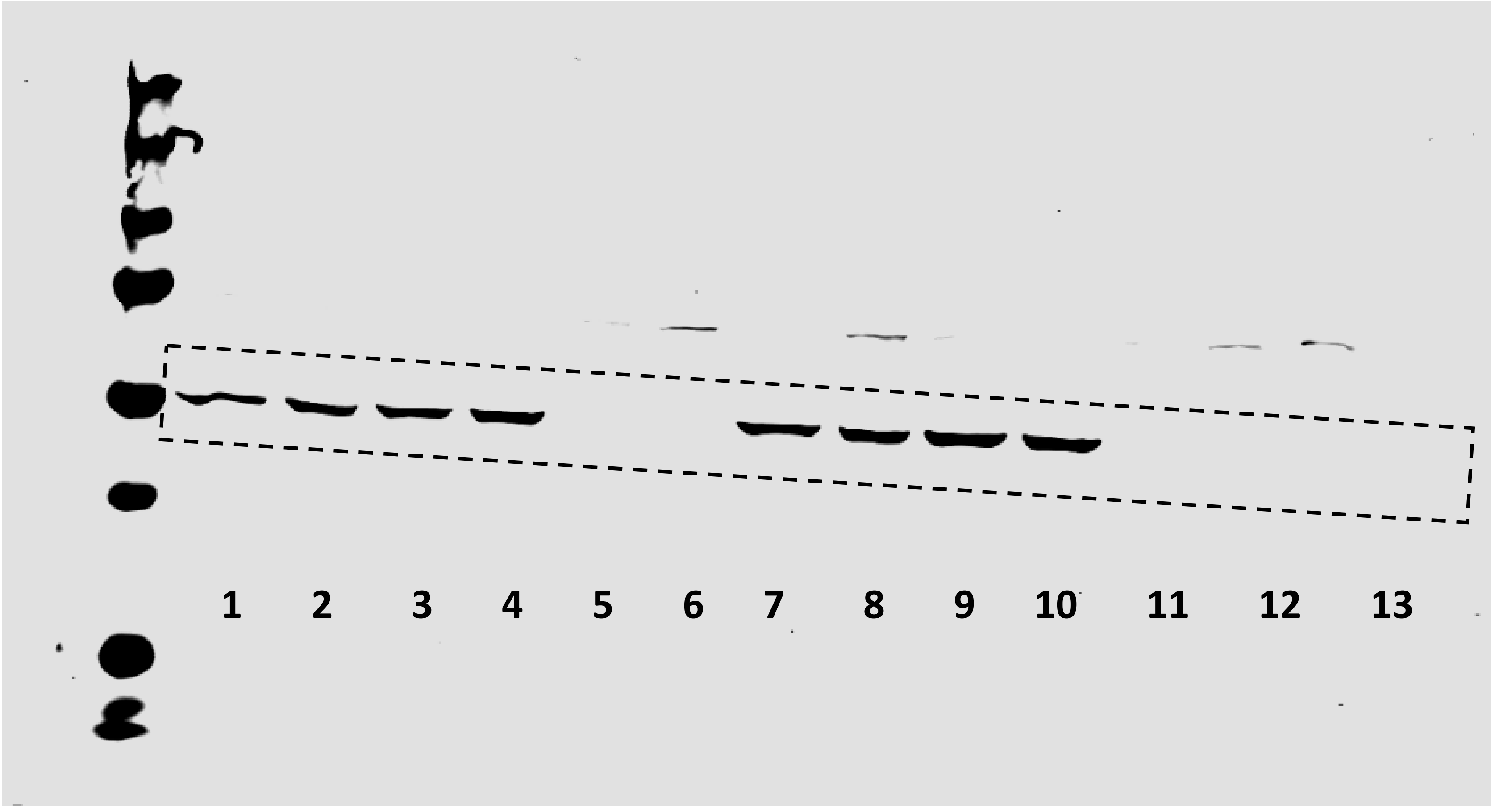

Anti TPH2

1-4: Tph2 HEK cells
5-8:Tph2+Dnajc12
9-12:Dnajc12
13: Vector

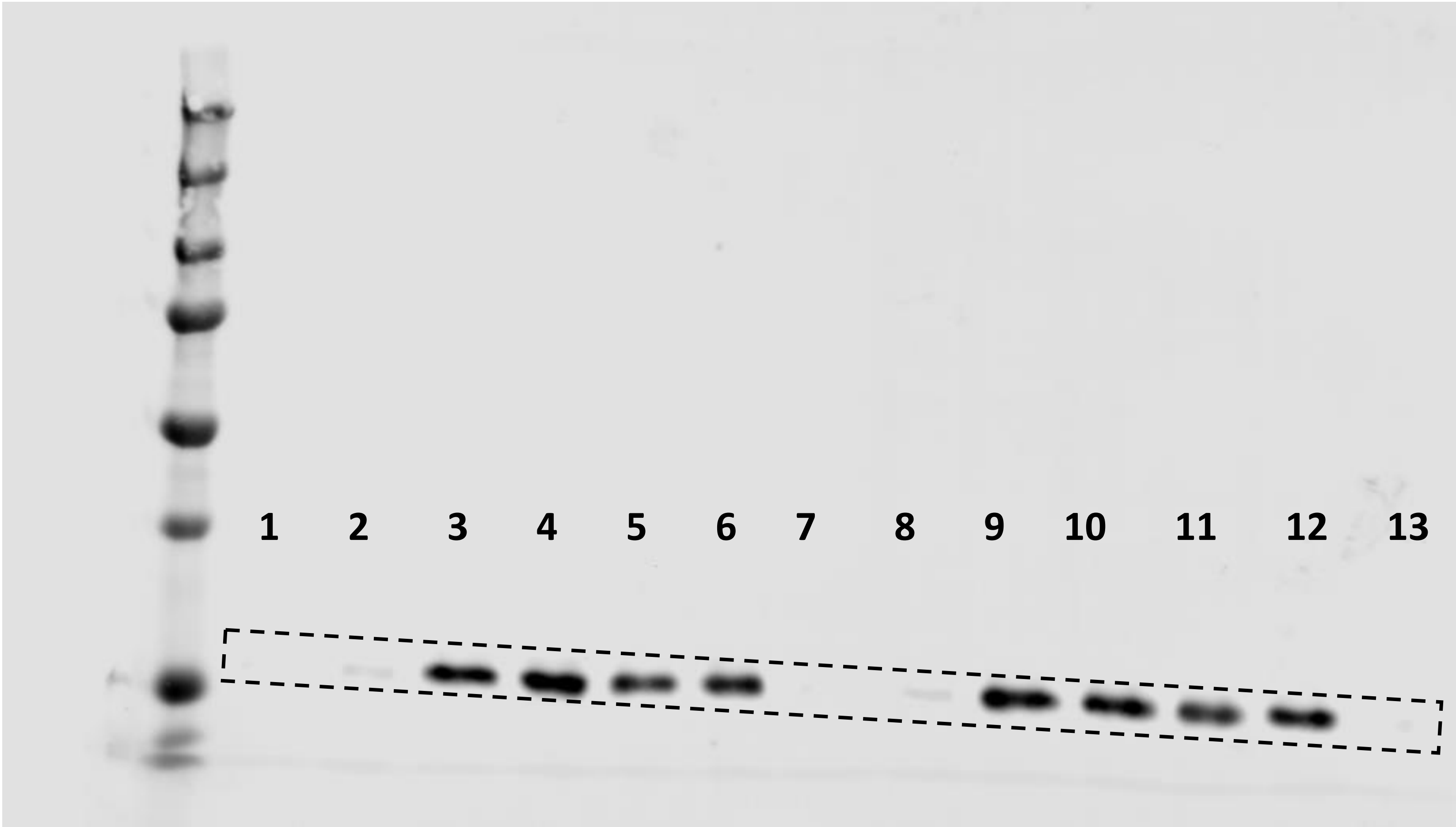

Anti Dnajc12

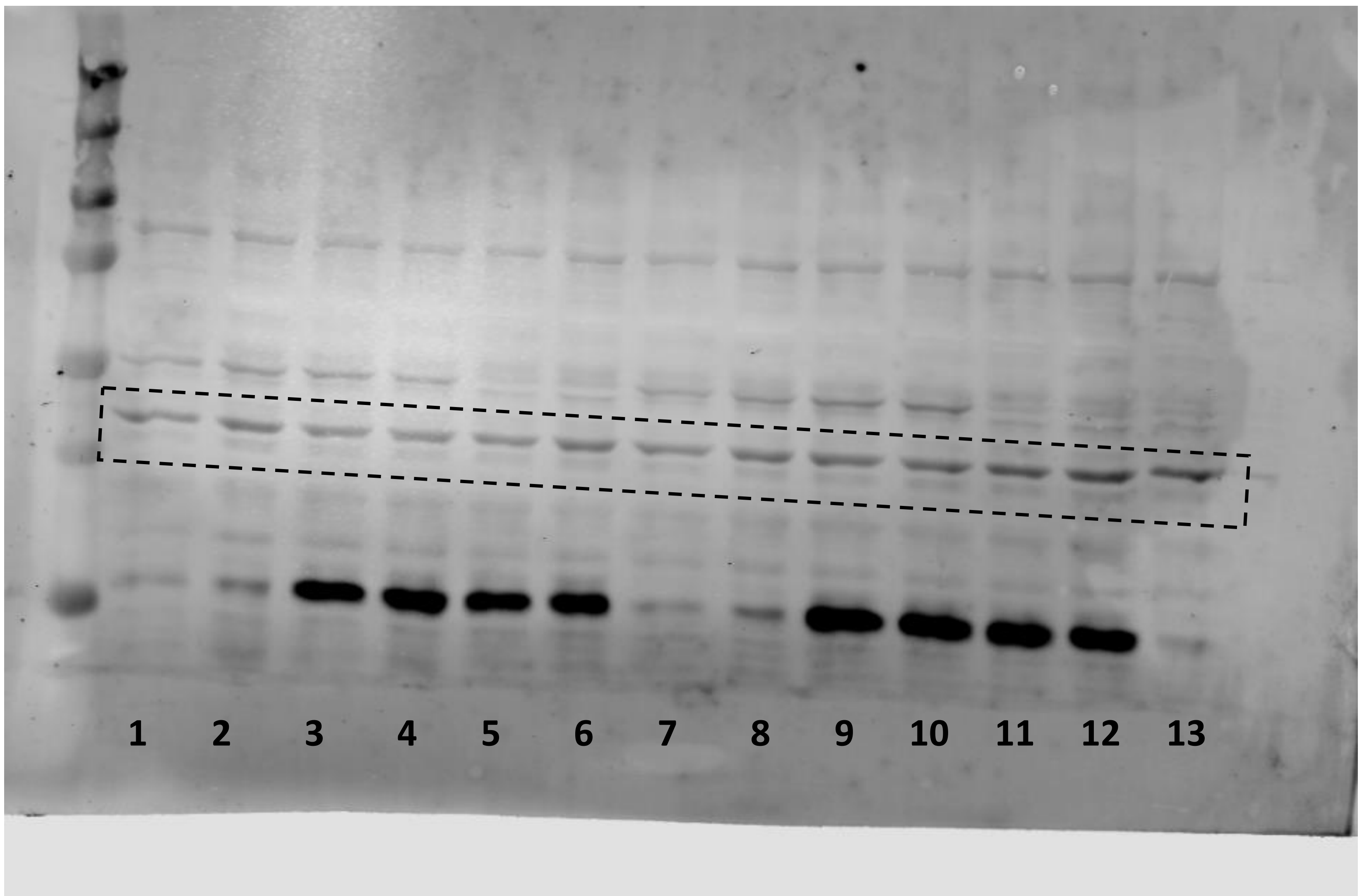

Anti GAPDH

Figure 4d

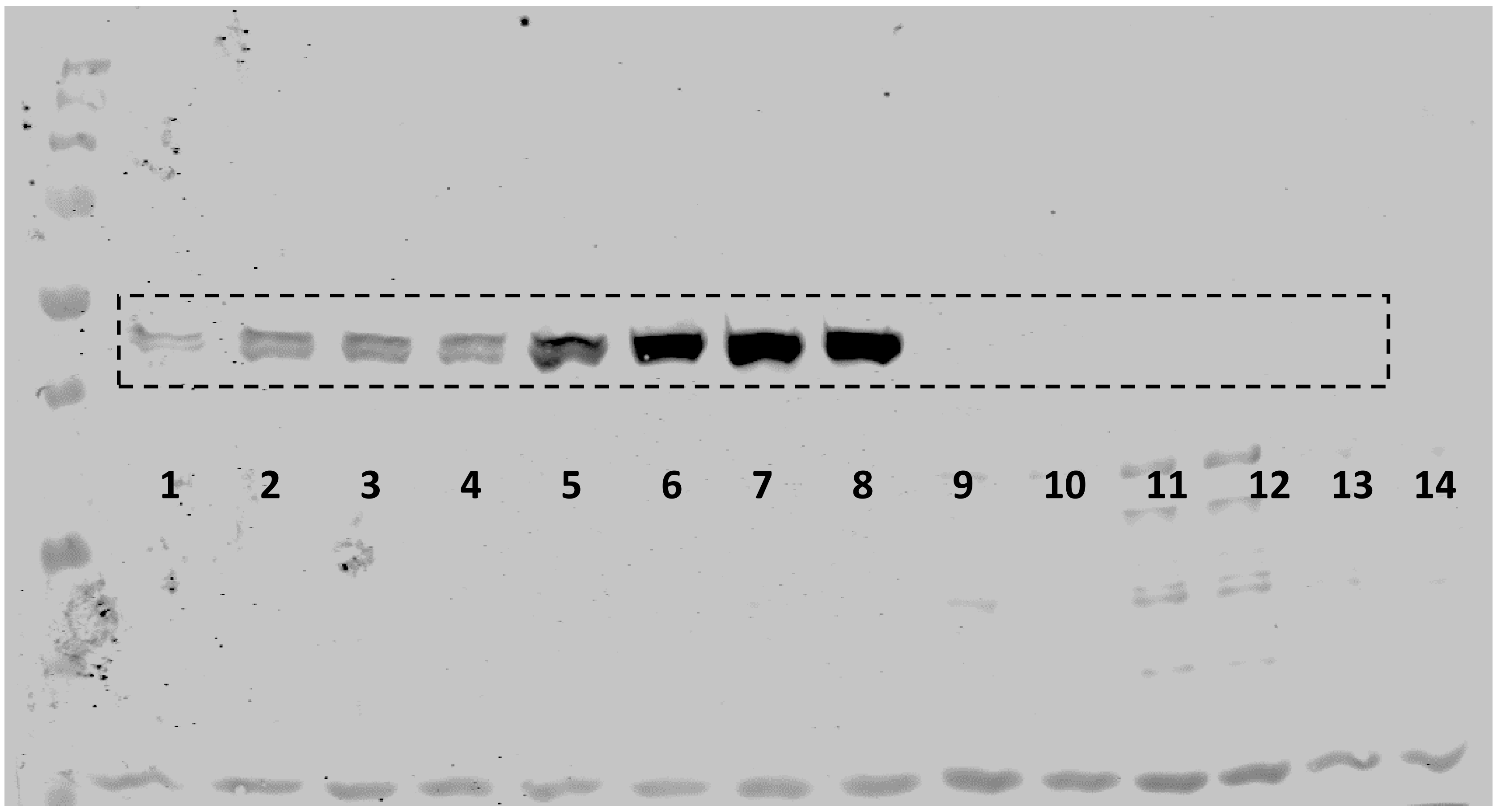

Anti TPH1

1-4: Tph1
5-8:Tph1+Dnajc12
9-12:Dnajc12
13-14: Vector

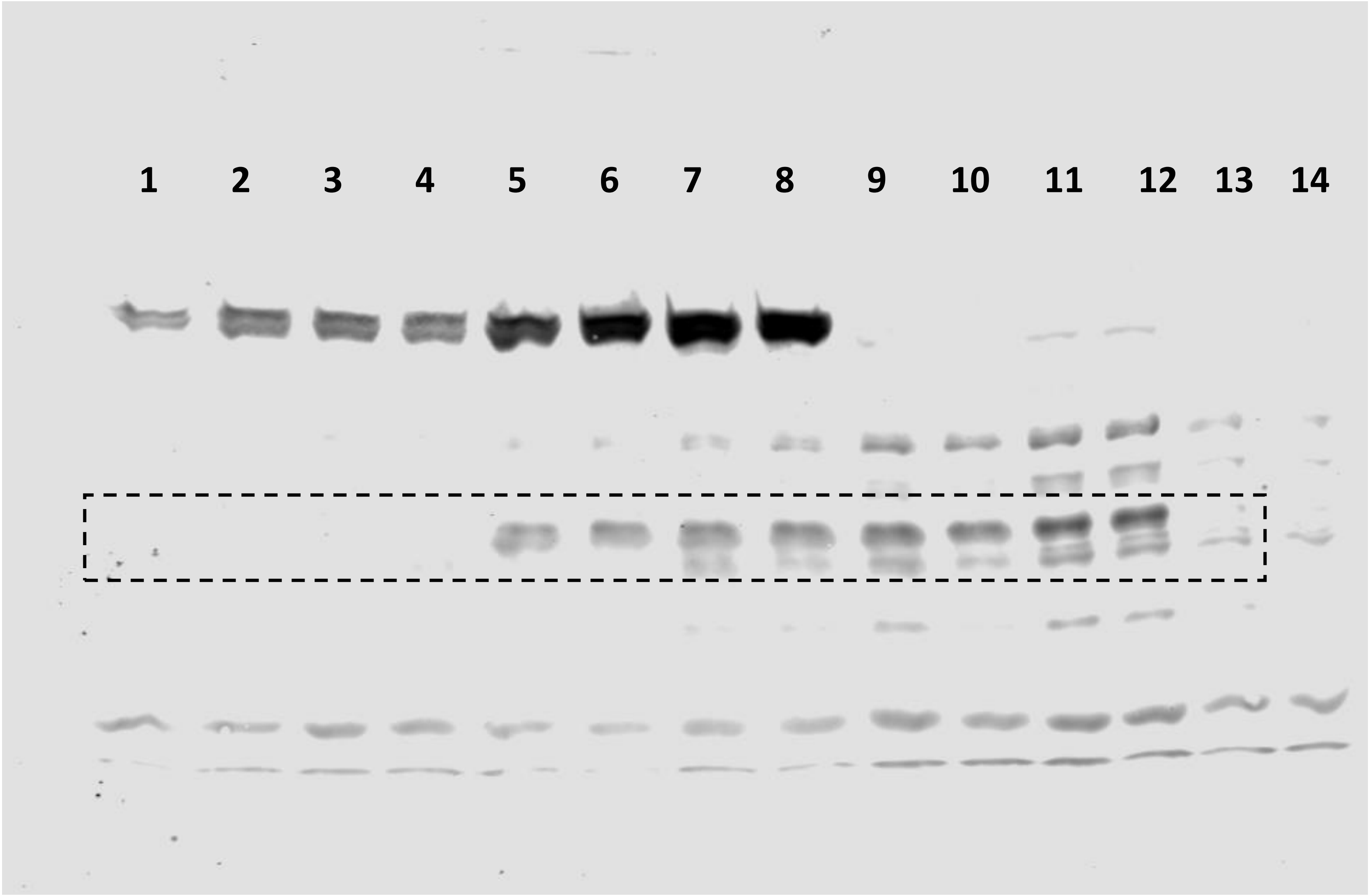

Anti DNAJC12

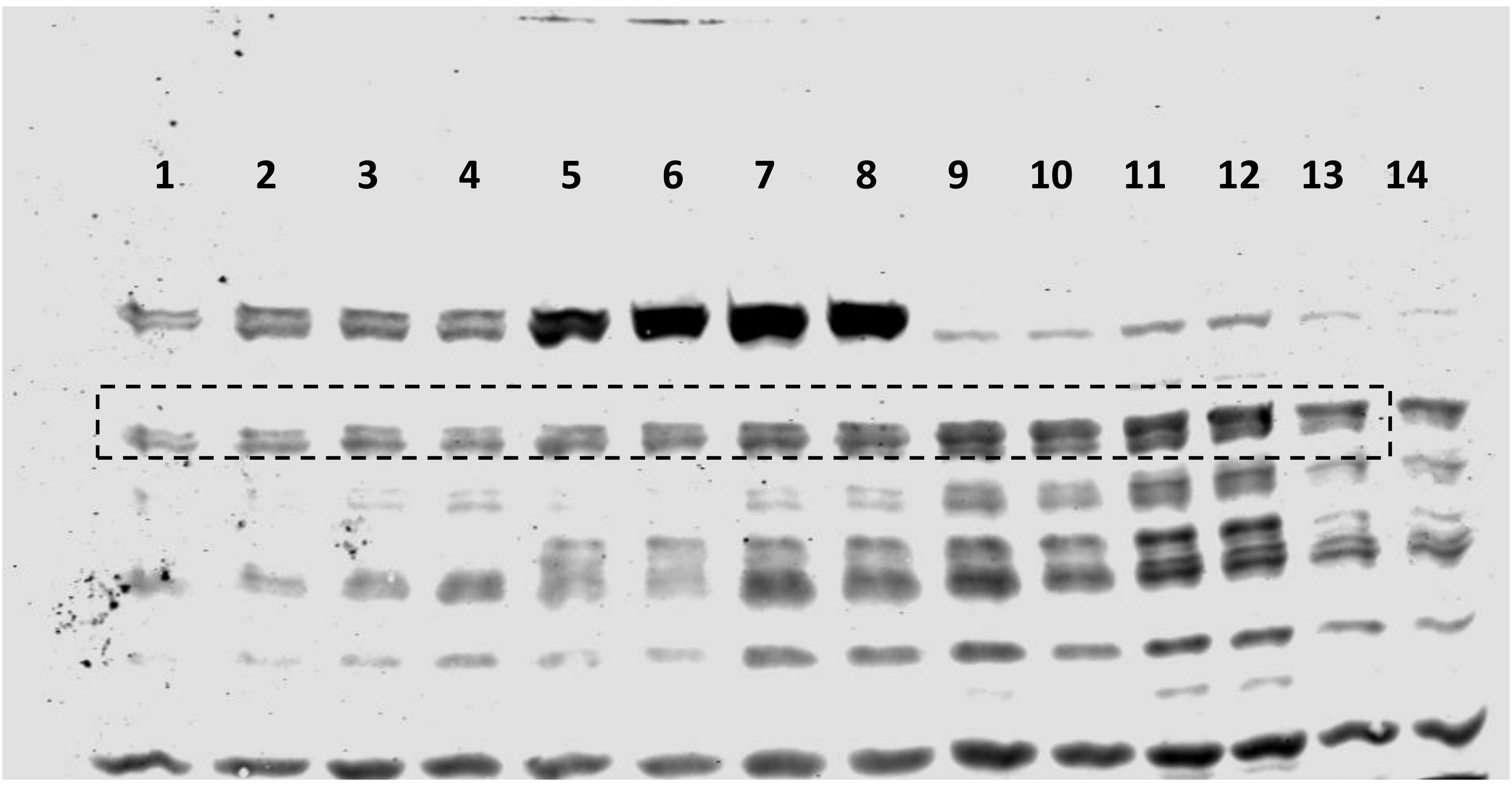

Anti GAPDH

Figure 5a upper panel

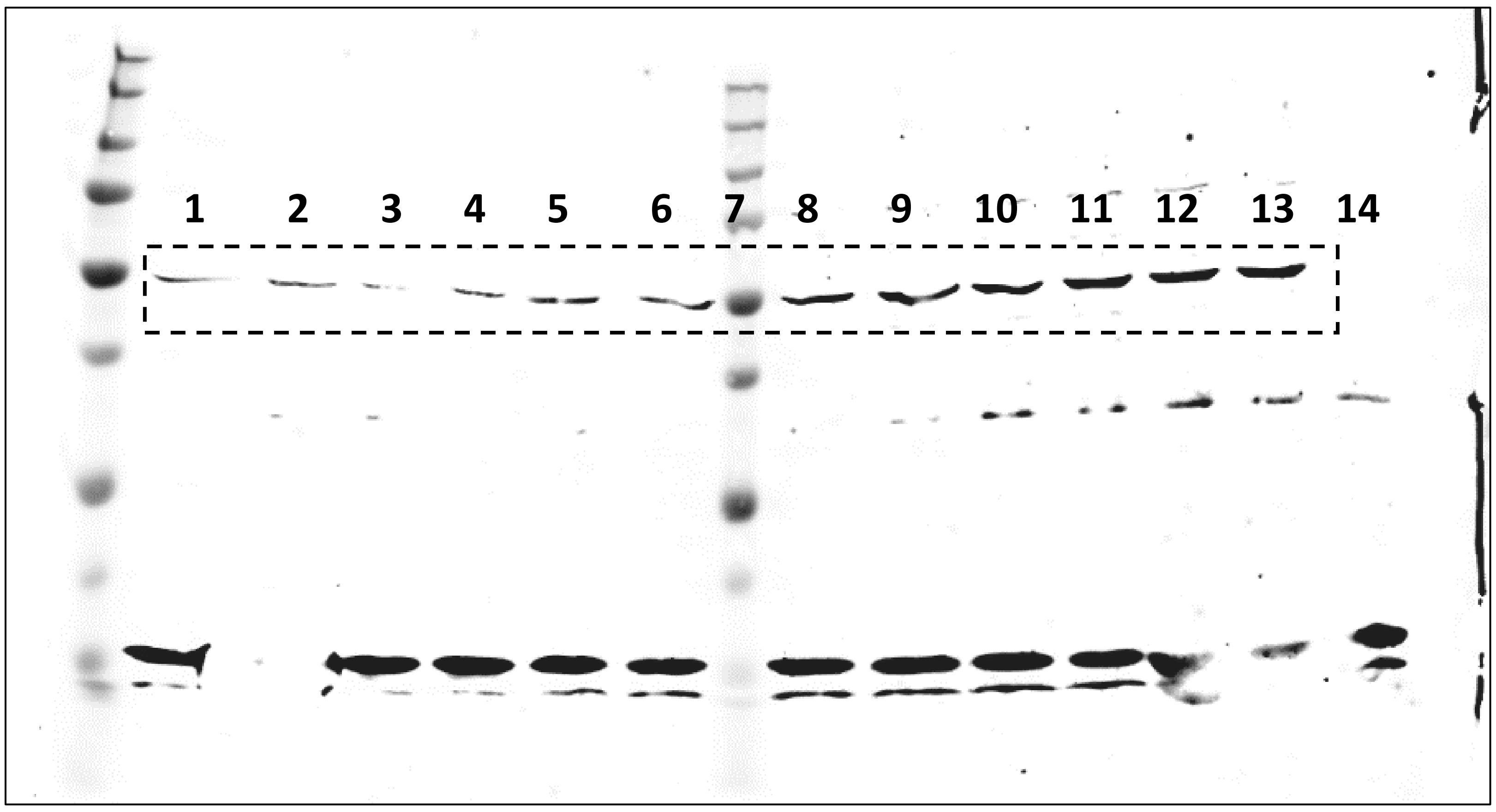

Anti TPH2

1-3: Tph2
4-6:Tph2 Mg132
7: marker
8-10:Tph2+Dnajc12
11-13: Tph2+Dnajc12 Mg132
14: Vector

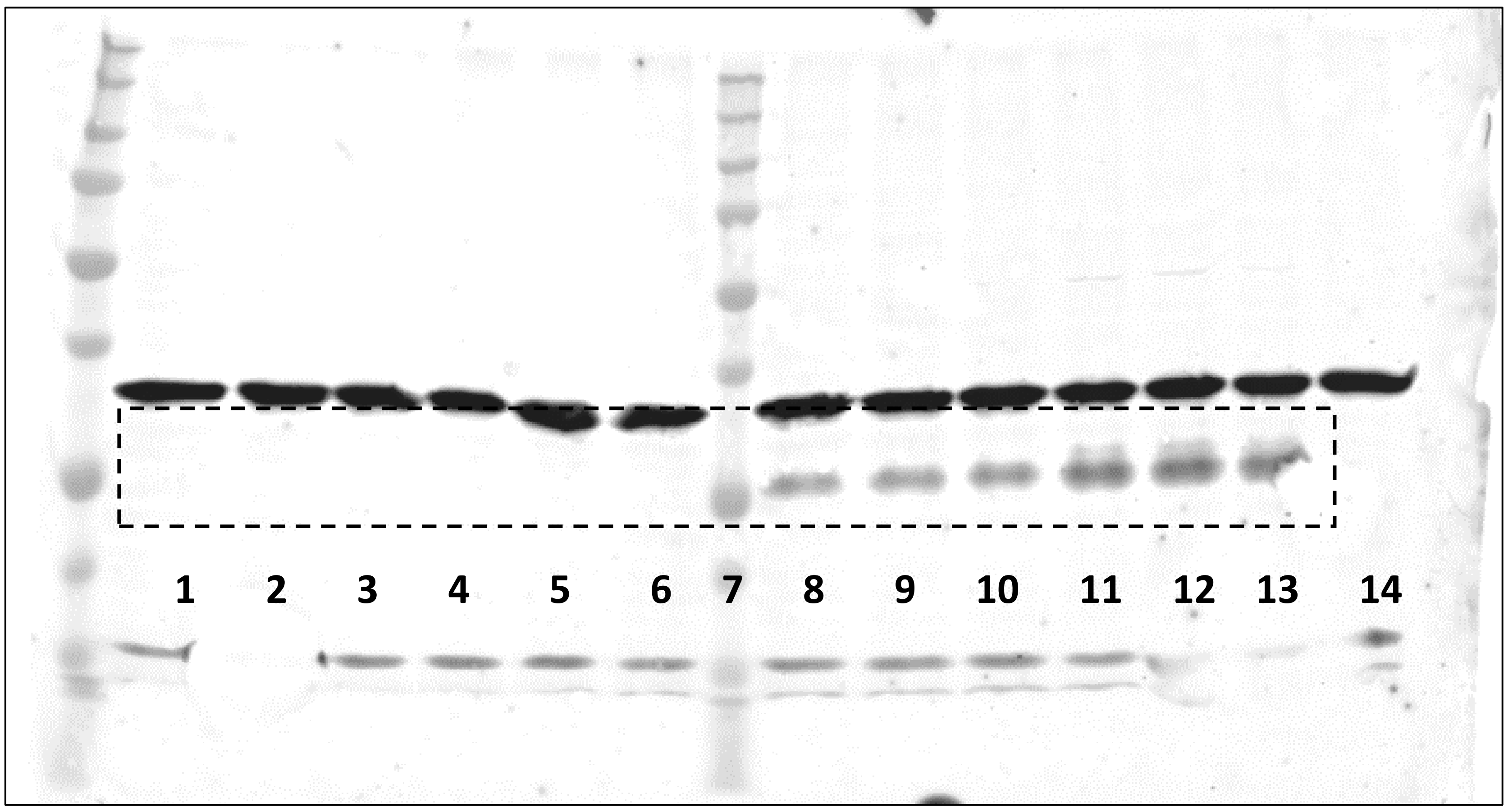

Anti DNAJC12

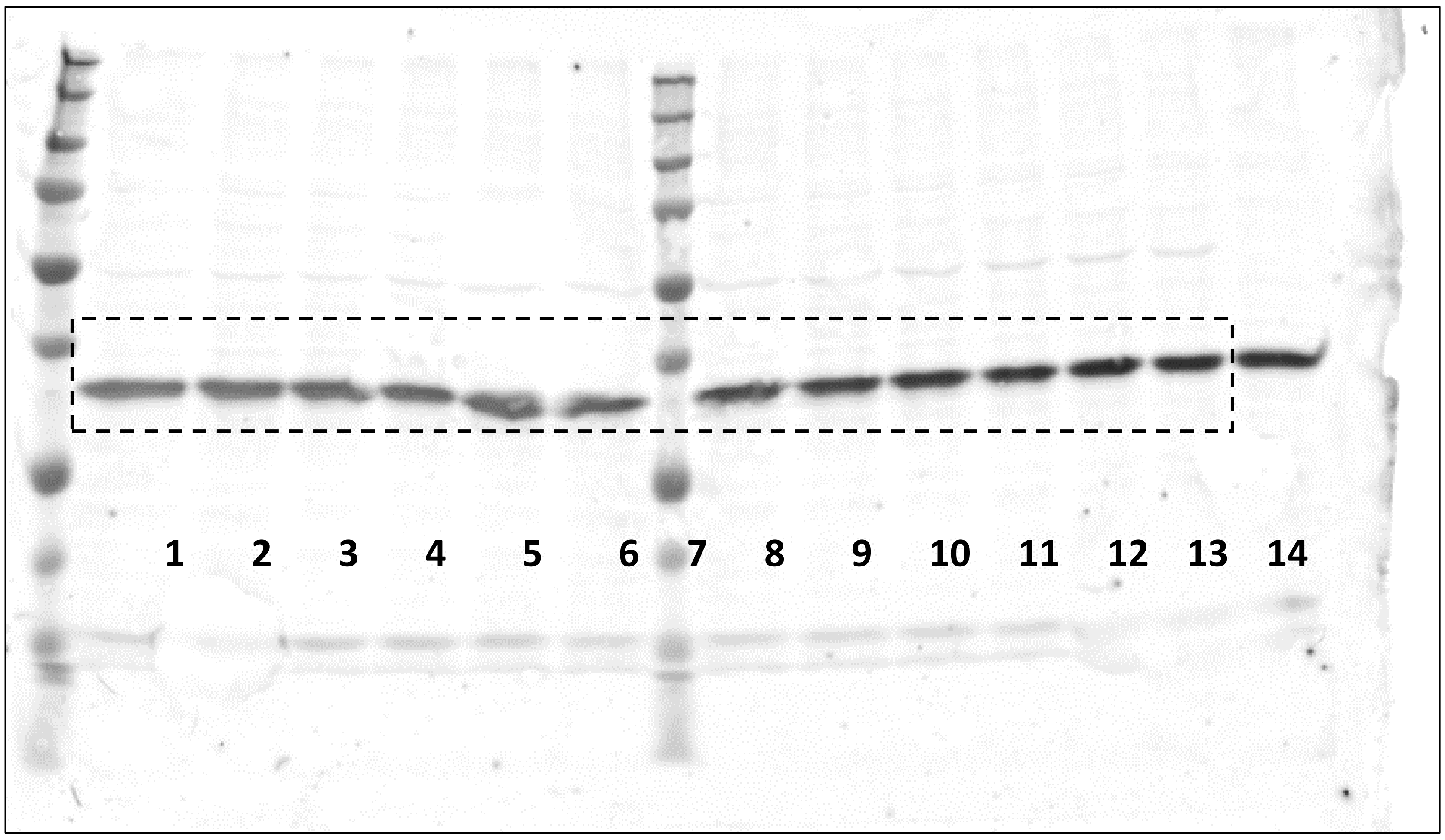

Anti GAPDH

Figure 5a lower panel

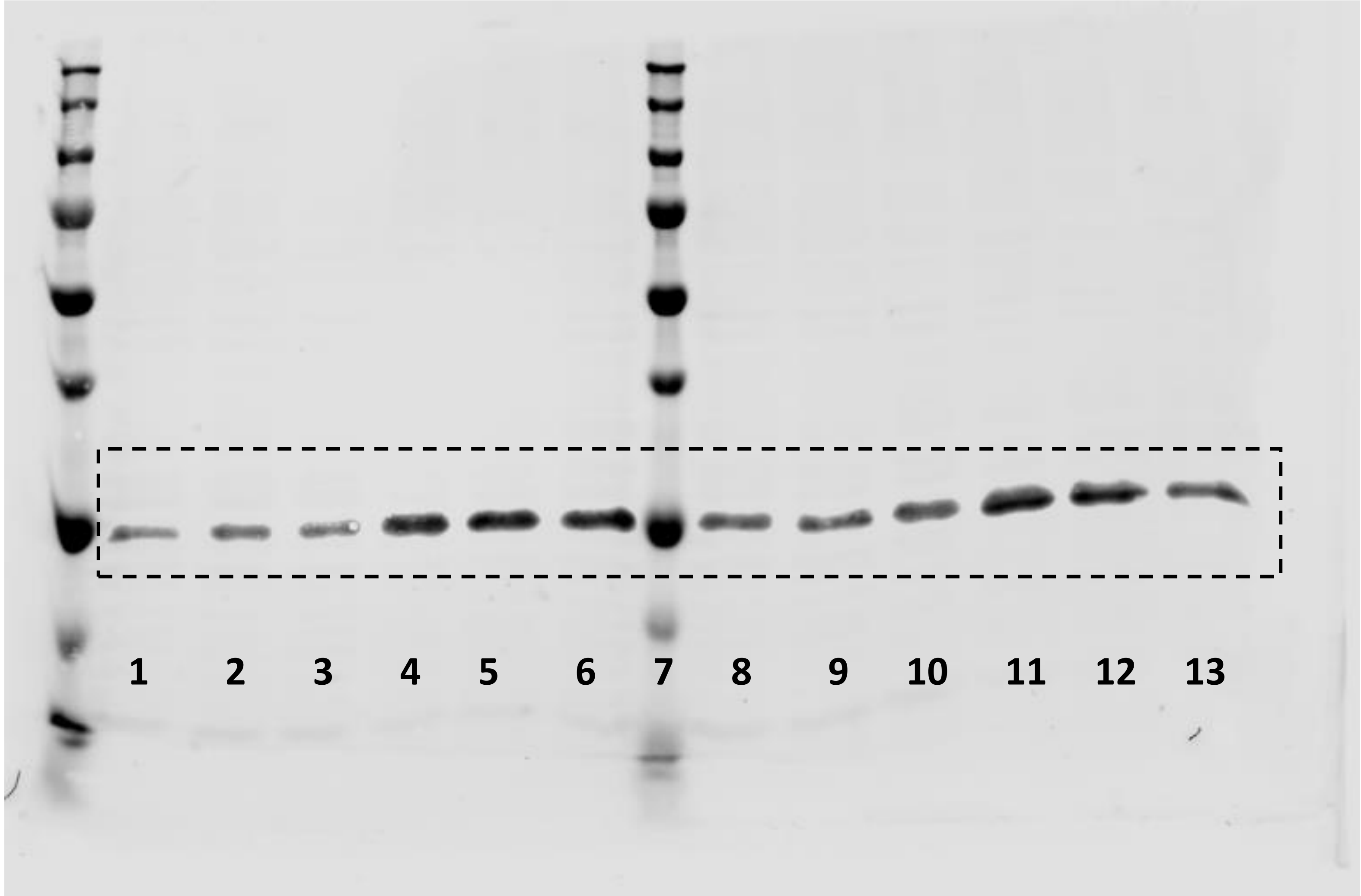

Anti DNAJC12

1-3: Dnajc12
4-6:DnaJc12 Mg132
7: marker
9-10: Tph2+Dnajc12
11-13: Tph2+Dnajc12 Mg132

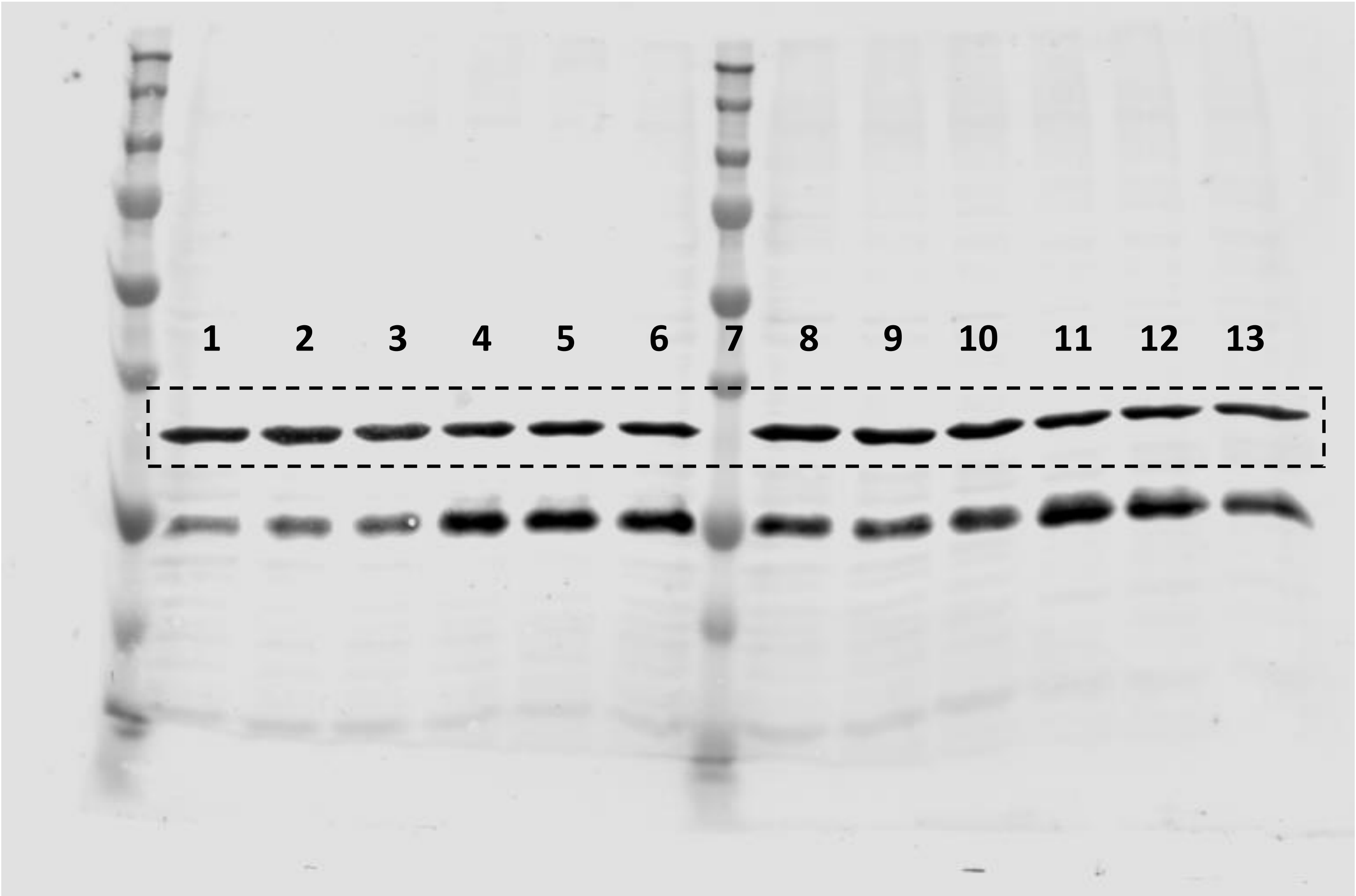

Anti GAPDH

Figure 5d upper panel

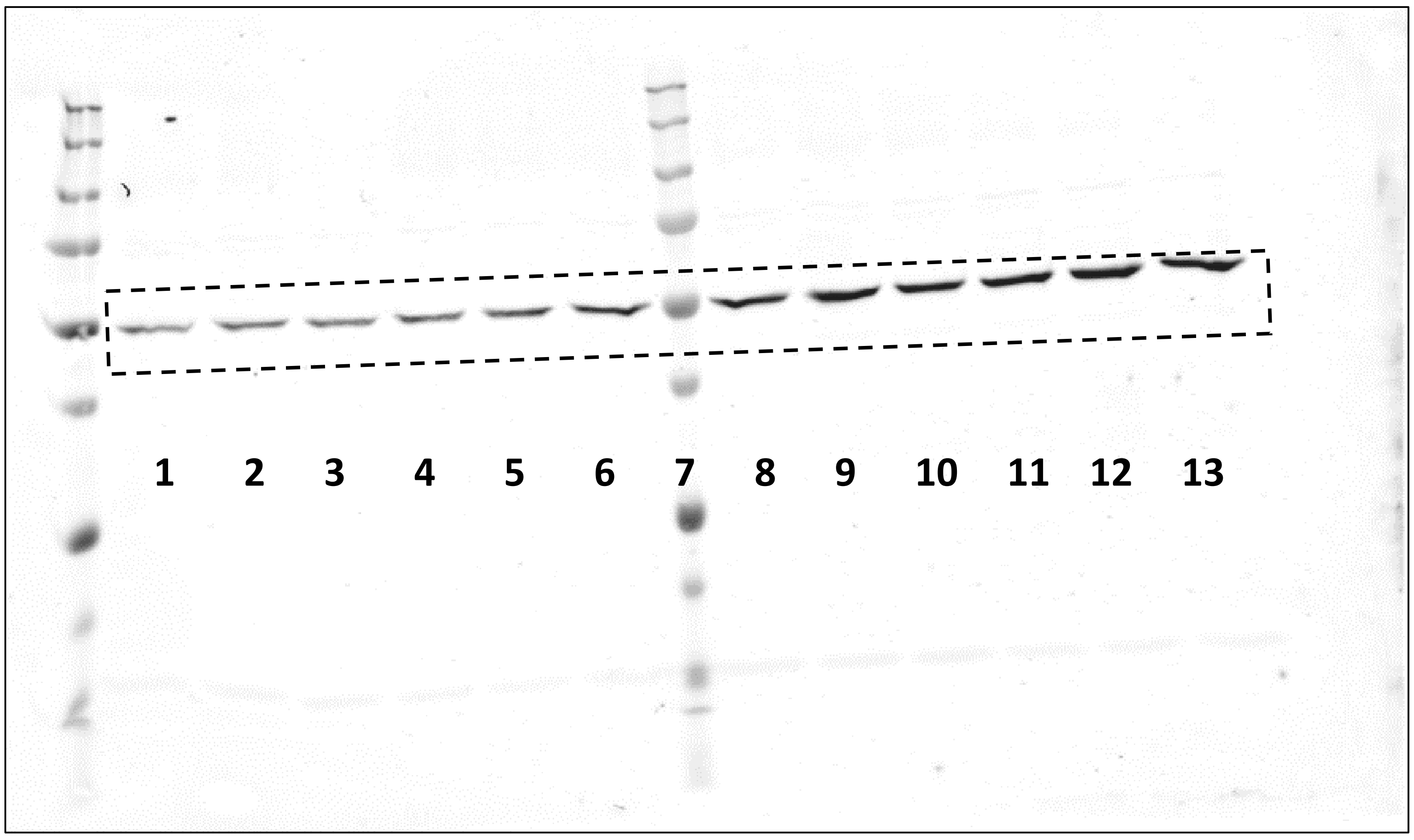

Anti TPH1

1-3: Tph1
4-6:Tph1 Mg132
7: marker
9-10: Tph1+Dnajc12
11-13: Tph1+Dnajc12 Mg132

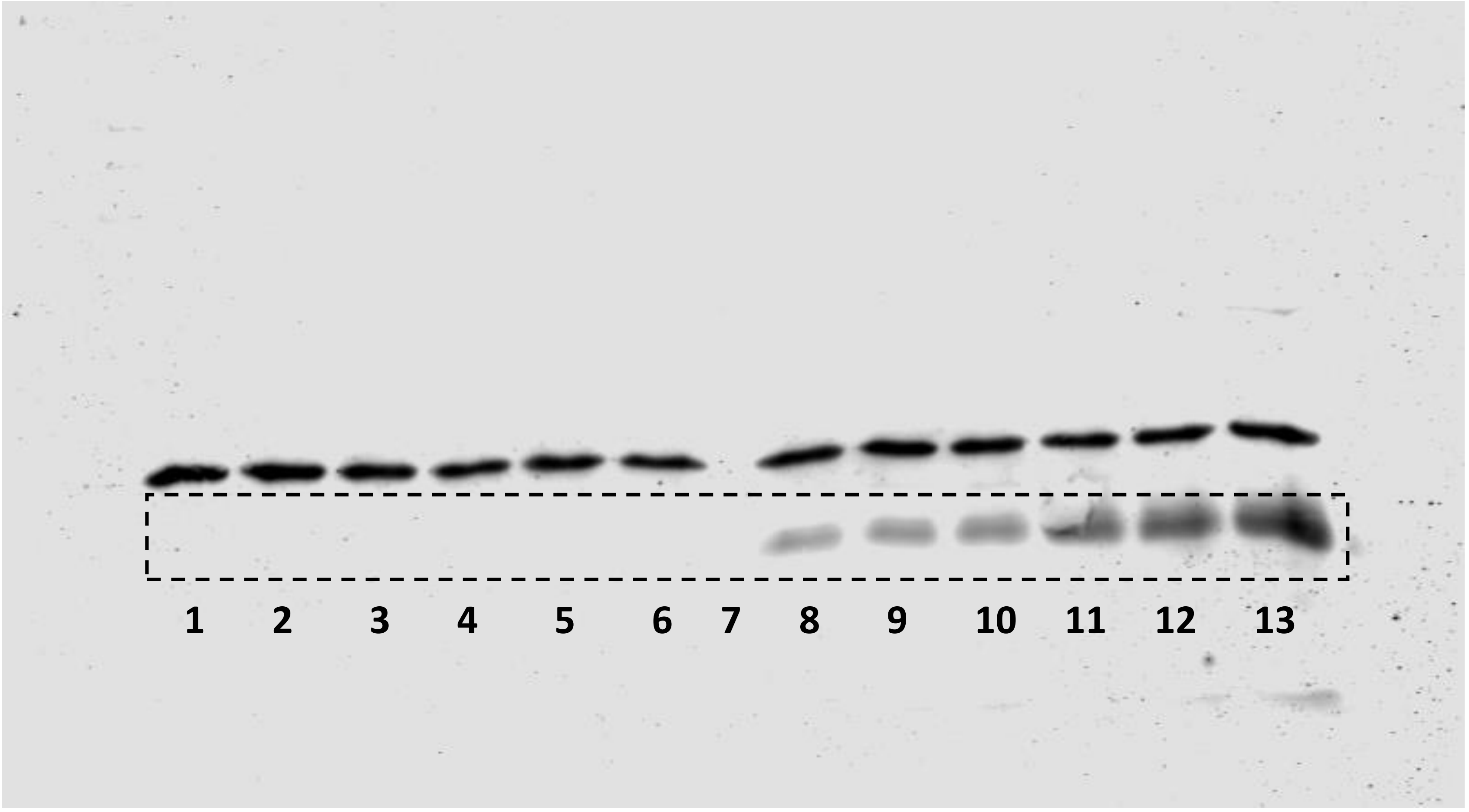

Anti DNAJC12

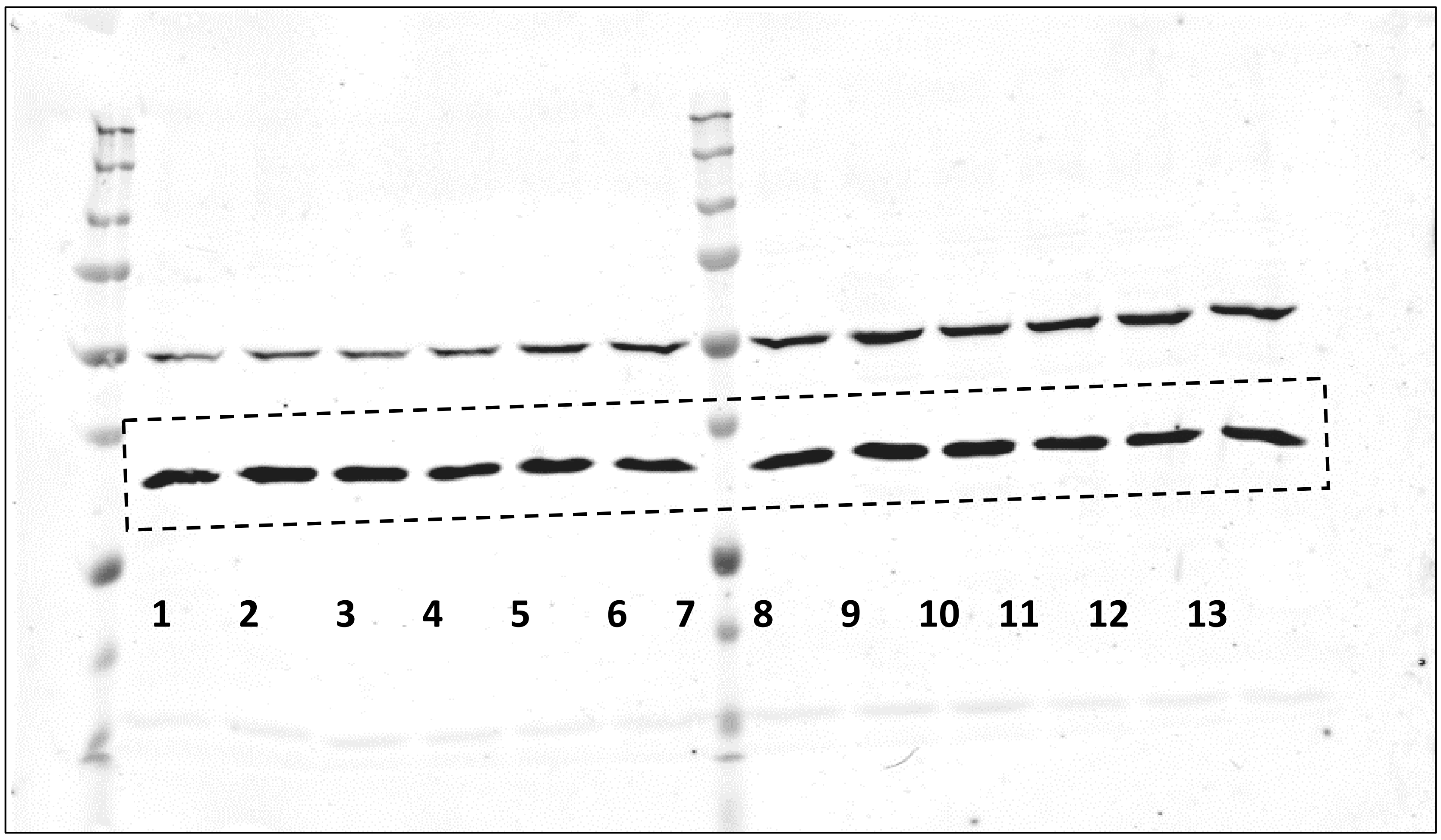

Anti GAPDH

Figure 5d lower panel

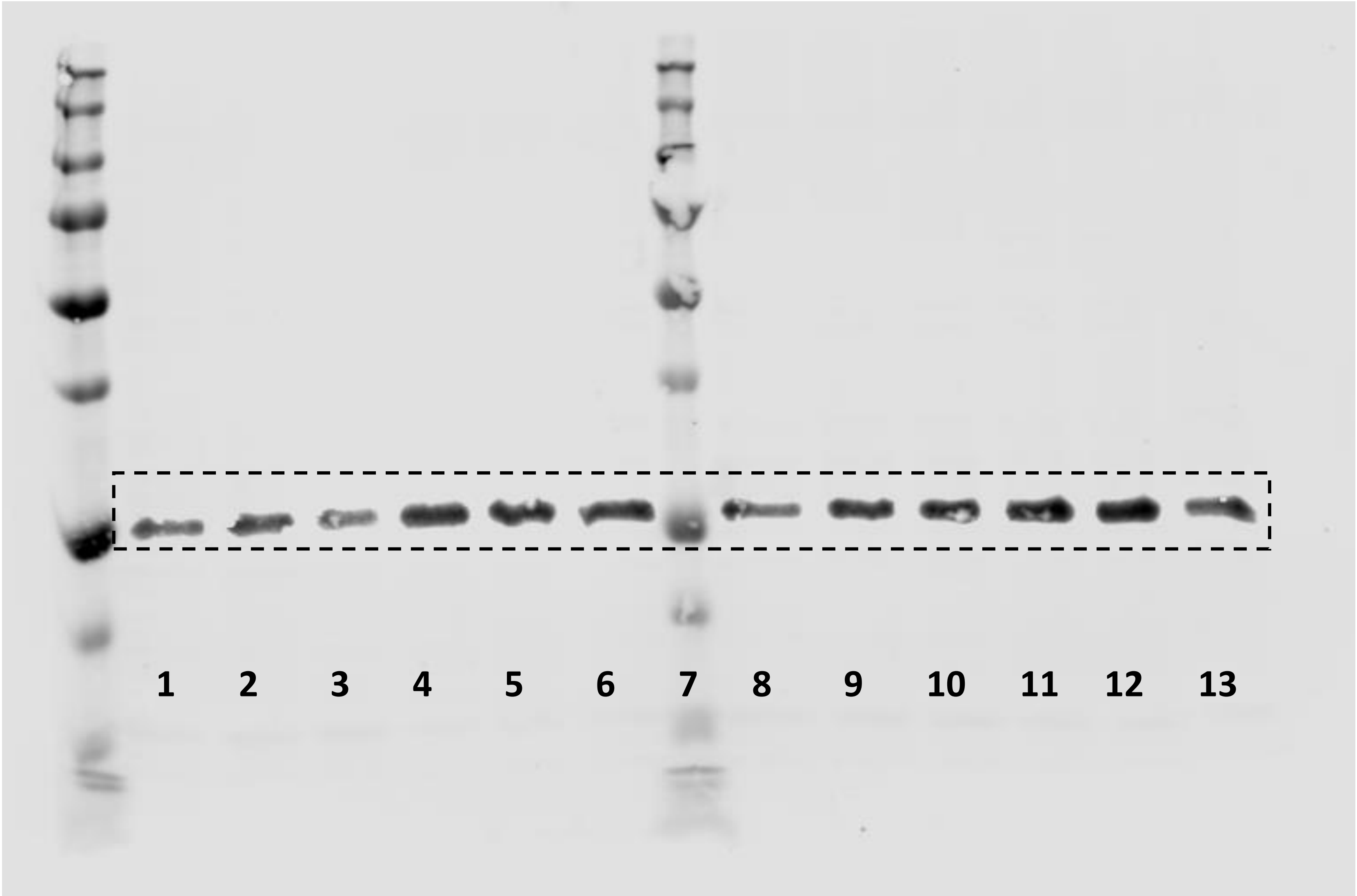

Anti DNAJC12

1-3: Dnajc12
4-6:DnaJc12 Mg132
7: marker
9-10: Tph1+Dnajc12
11-13: Tph1+Dnajc12 Mg132

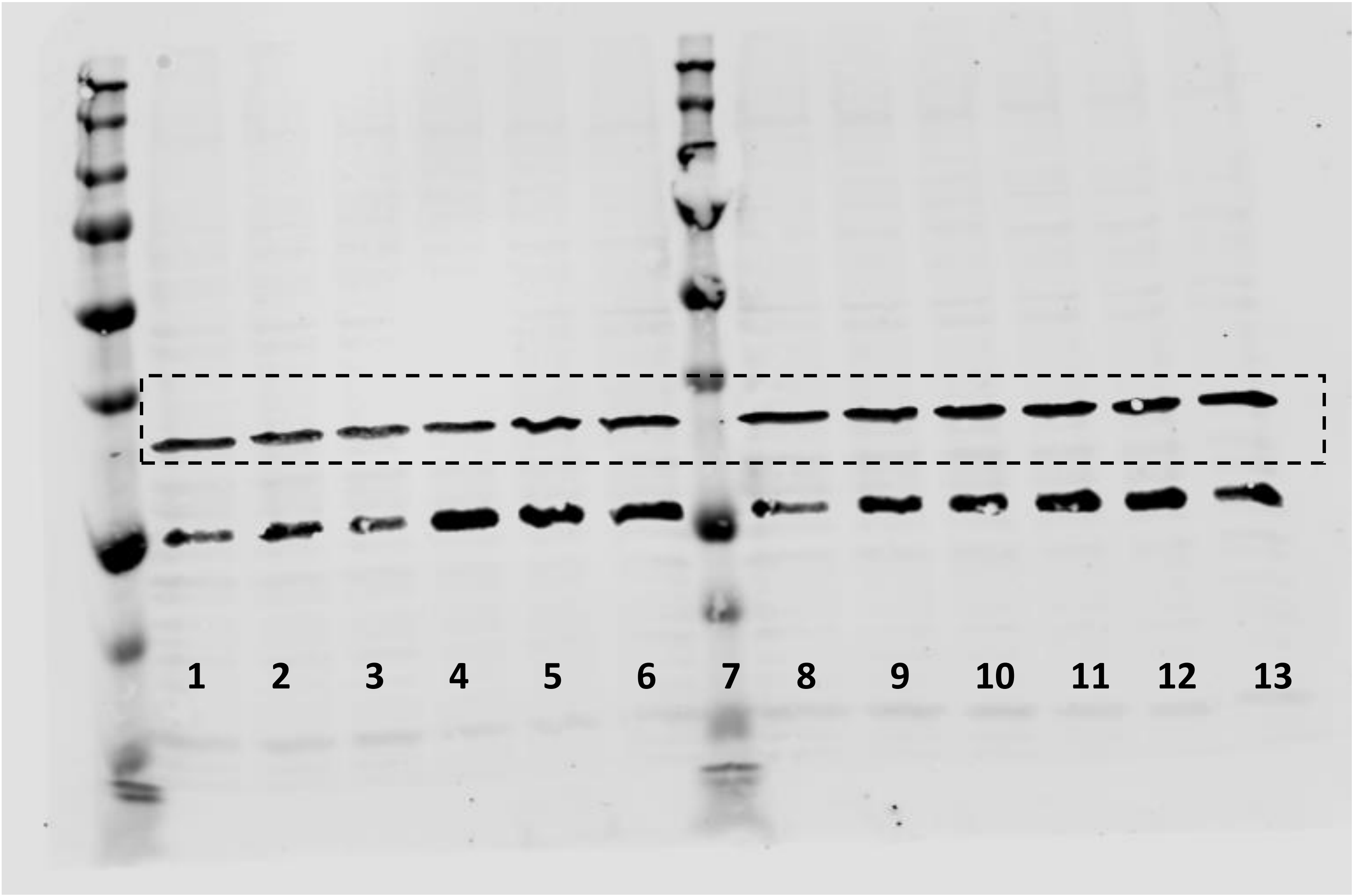

Anti GAPDH

Figure 6a

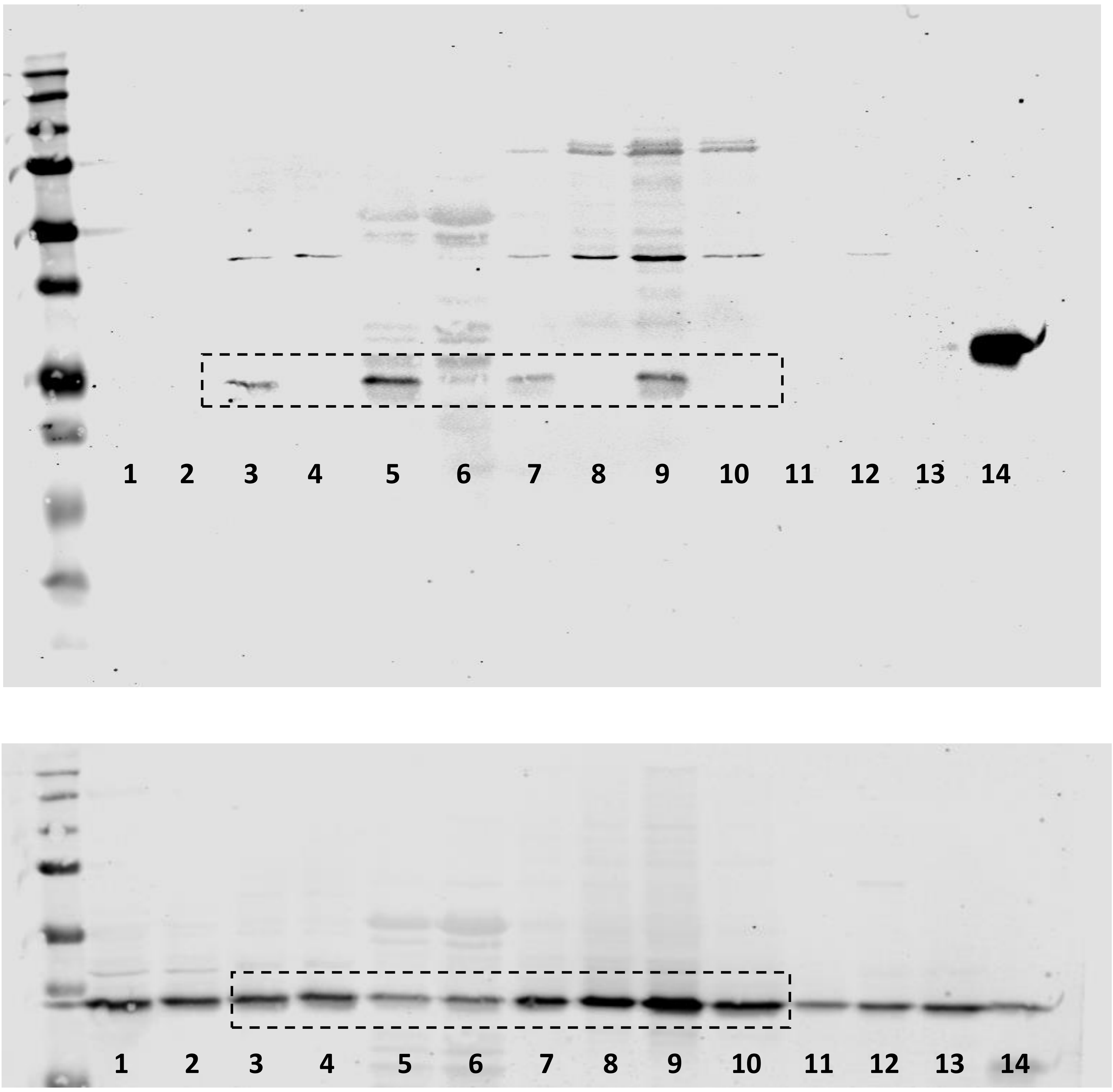

1: WT duodenum
2: DNAJC12 KO duodnum
3: WT kidney
4: DNAJC12 KO kidney
5: WT pancreas
6: DNAJC12 KO pancreas
7: WT raphe
8: DNAJC12 KO raphe
9: WT cerebellum
10: DNAJC12 KO cerebellum
11: WT pituitary,
12: WT adrenals
13: WT spleen
14: HEK cells transfected with *DNAJC12-FLAG*

Figure 6b

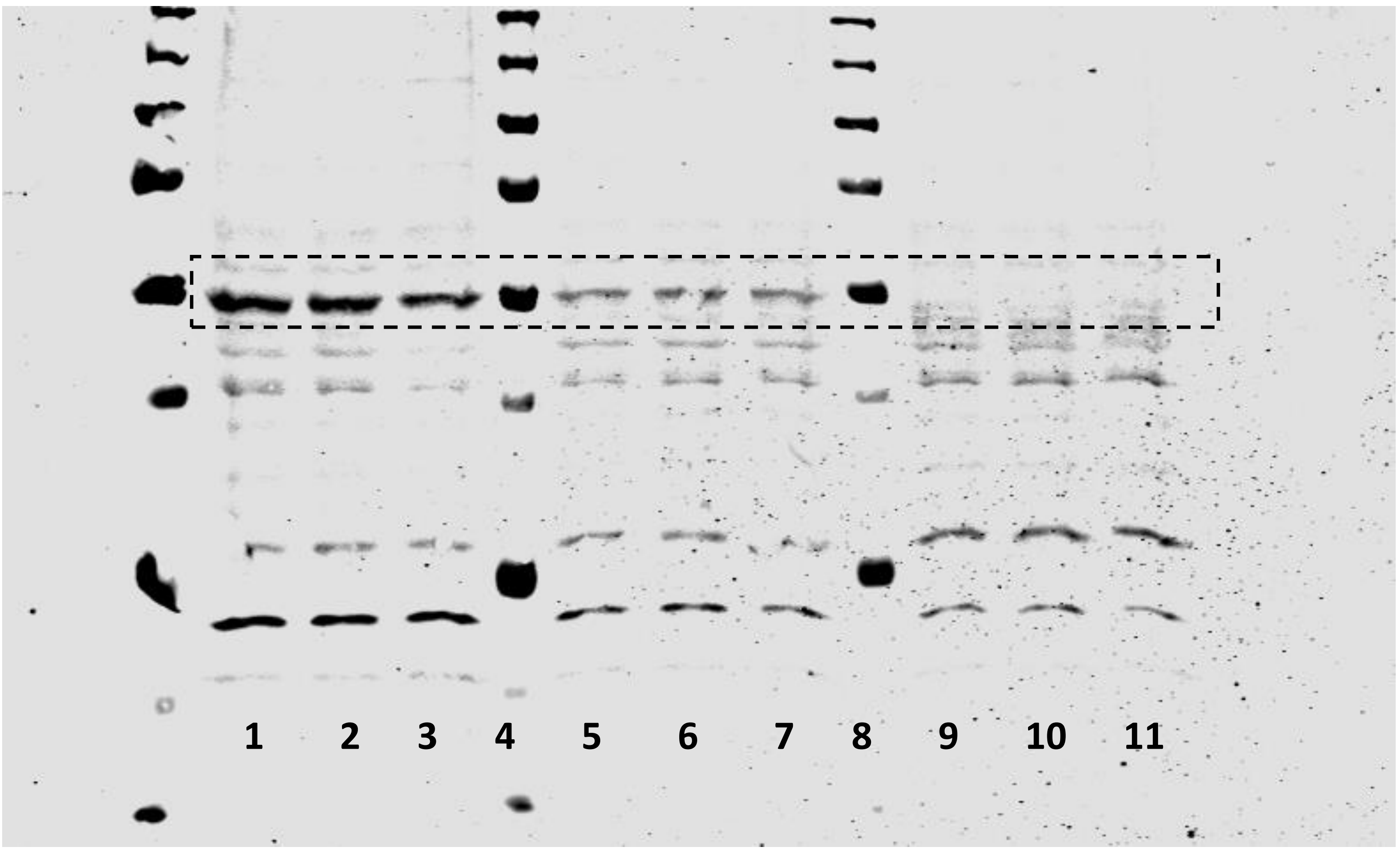

Anti TPH2

1-3: WT raphe
4: marker
5-7: DNAJC12 KO raphe
8: marker
9-11: TPH2 KO raphe

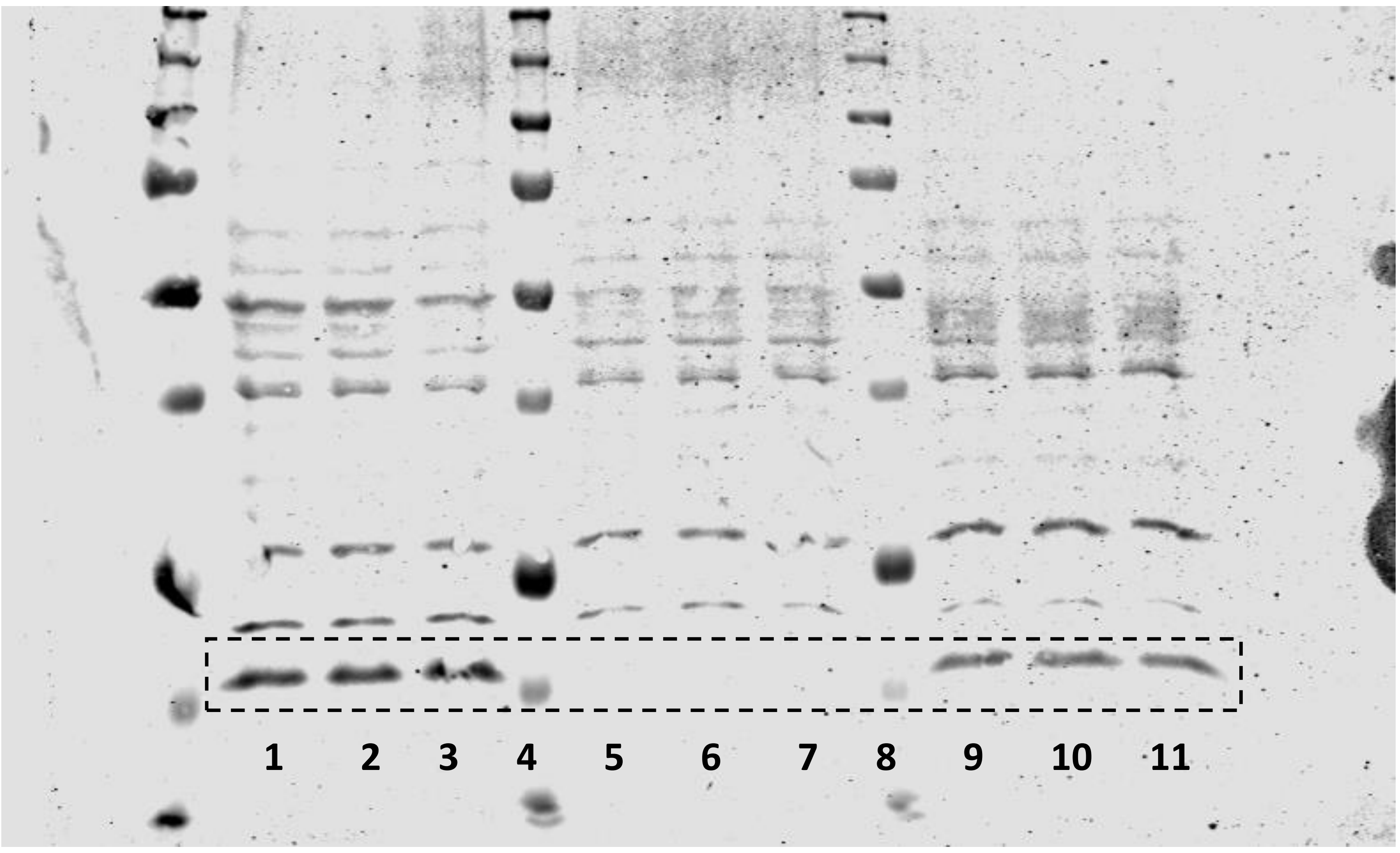

Anti DNAJC12

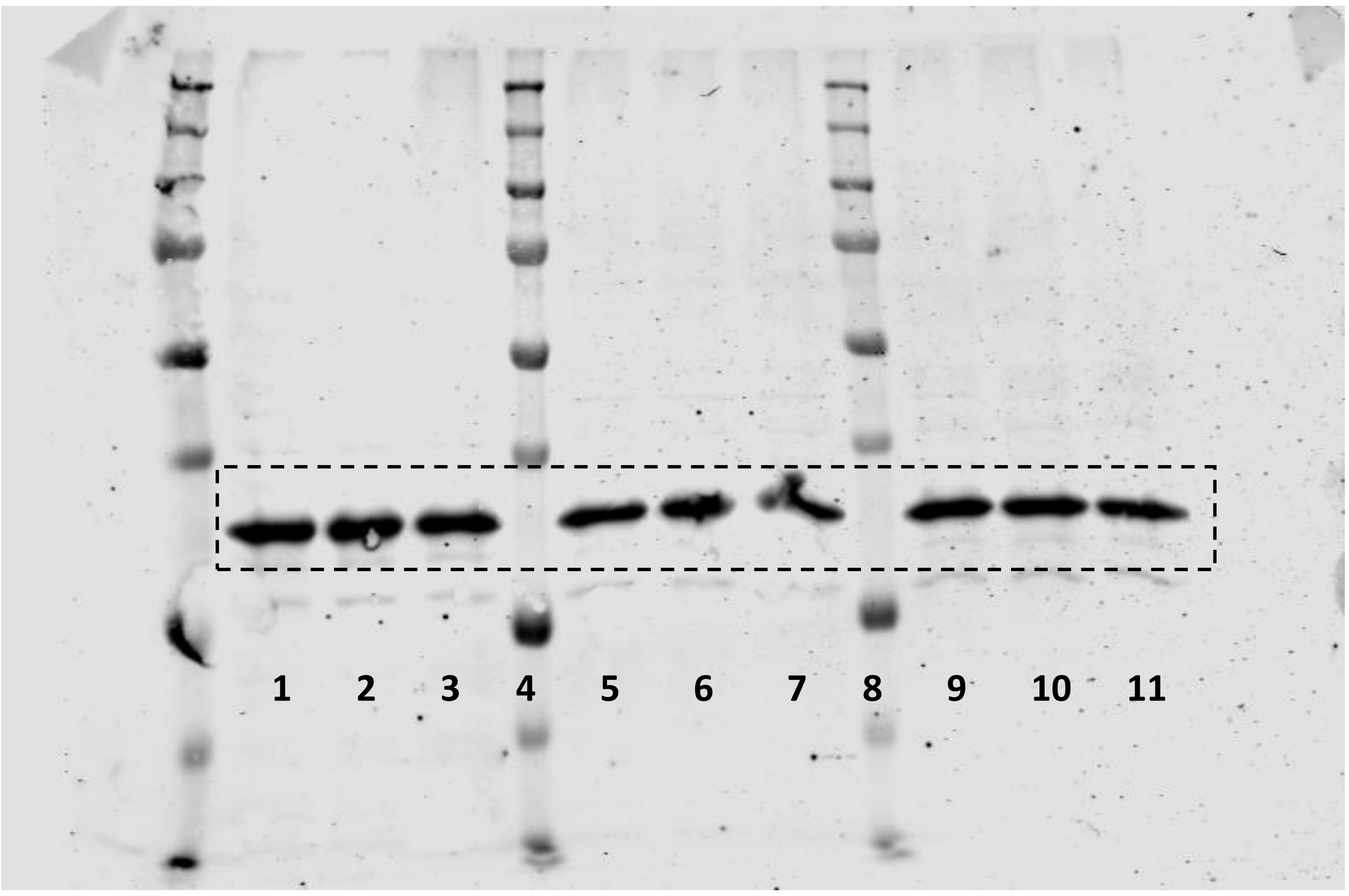

Anti GAPDH

Figure 6e

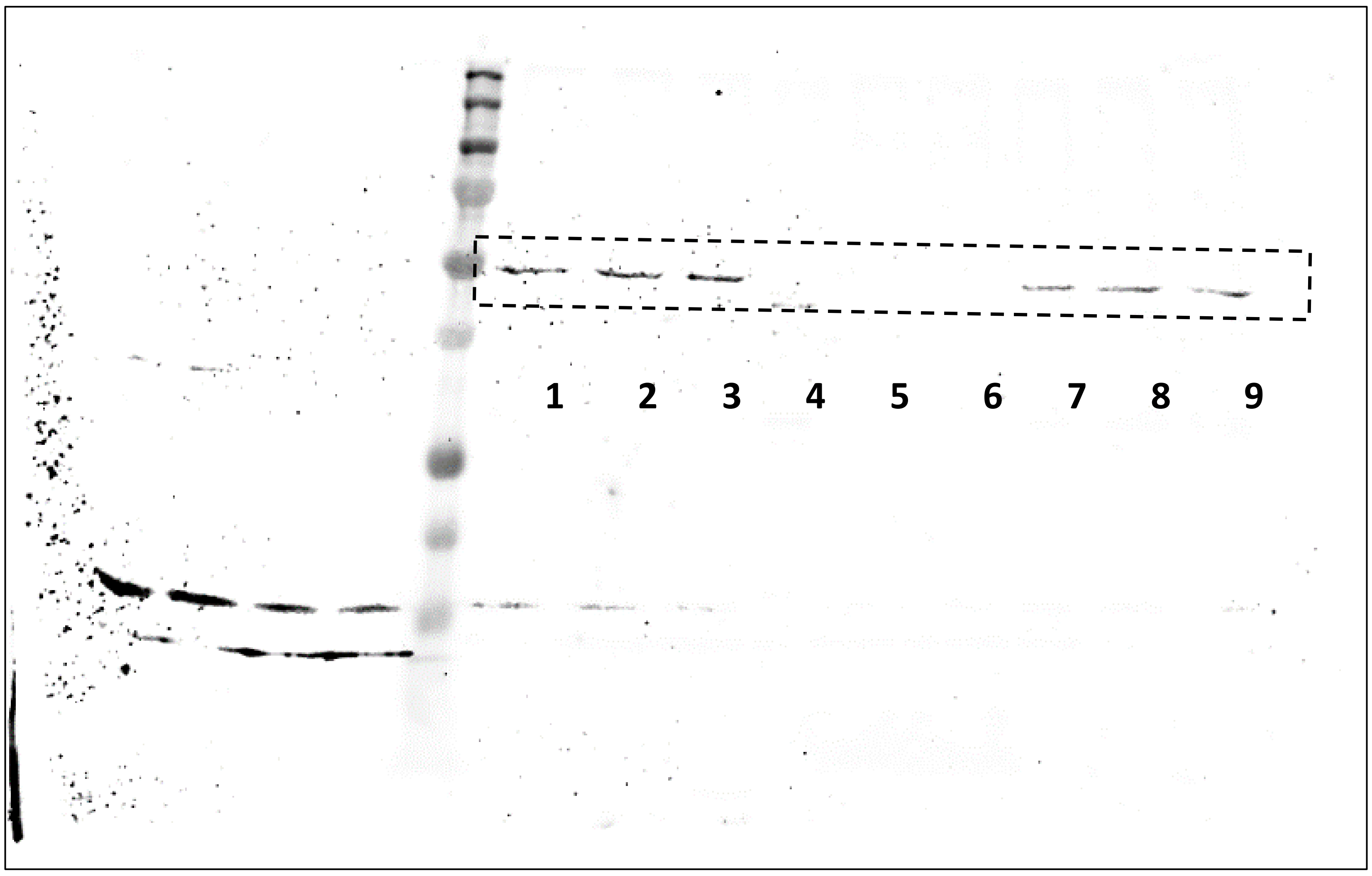

Anti TPH1

1-3: WT pineal
4-6: TPH1 KO pineal
7-9: DNAJC12 KO pineal

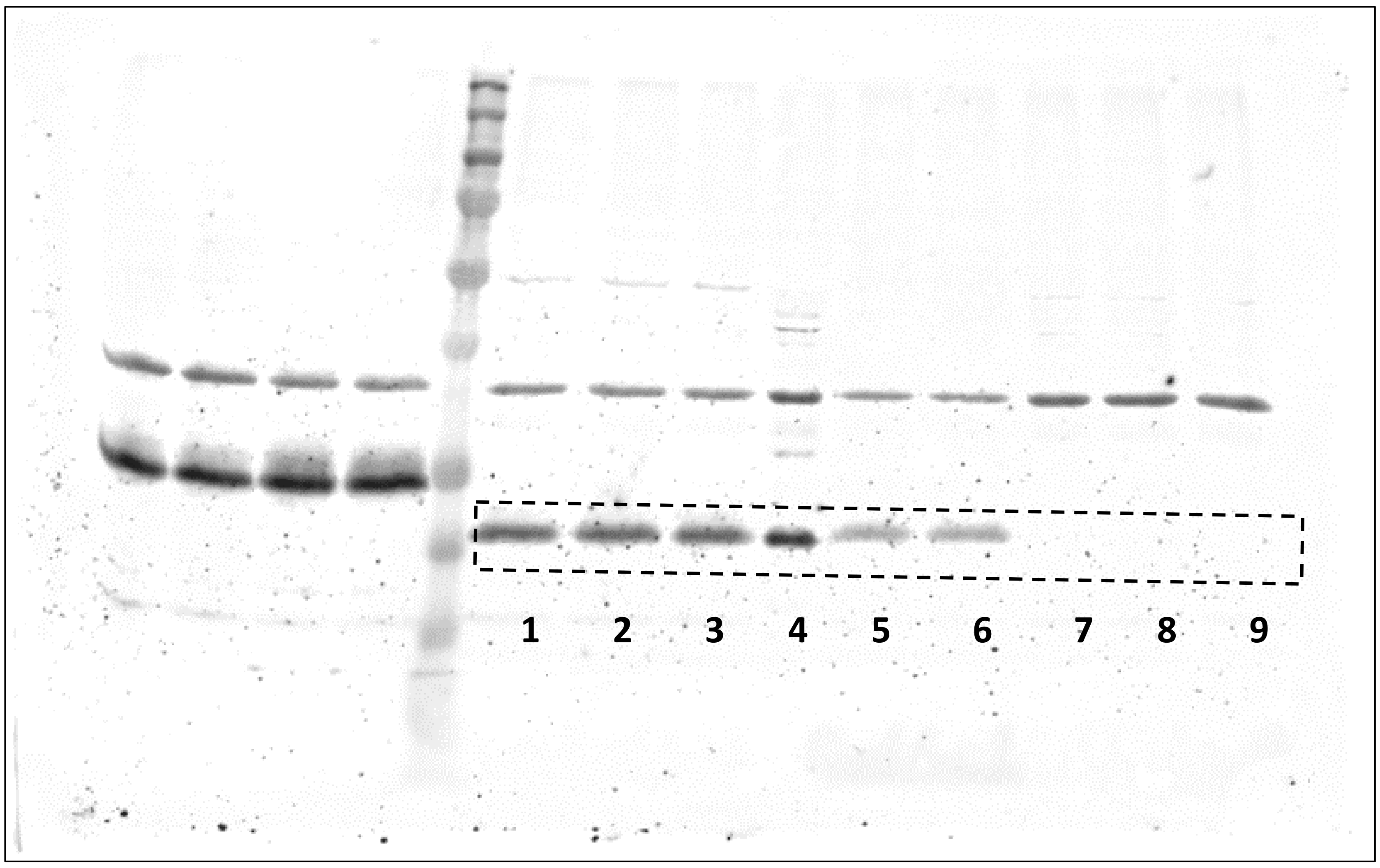

Anti DNAJC12

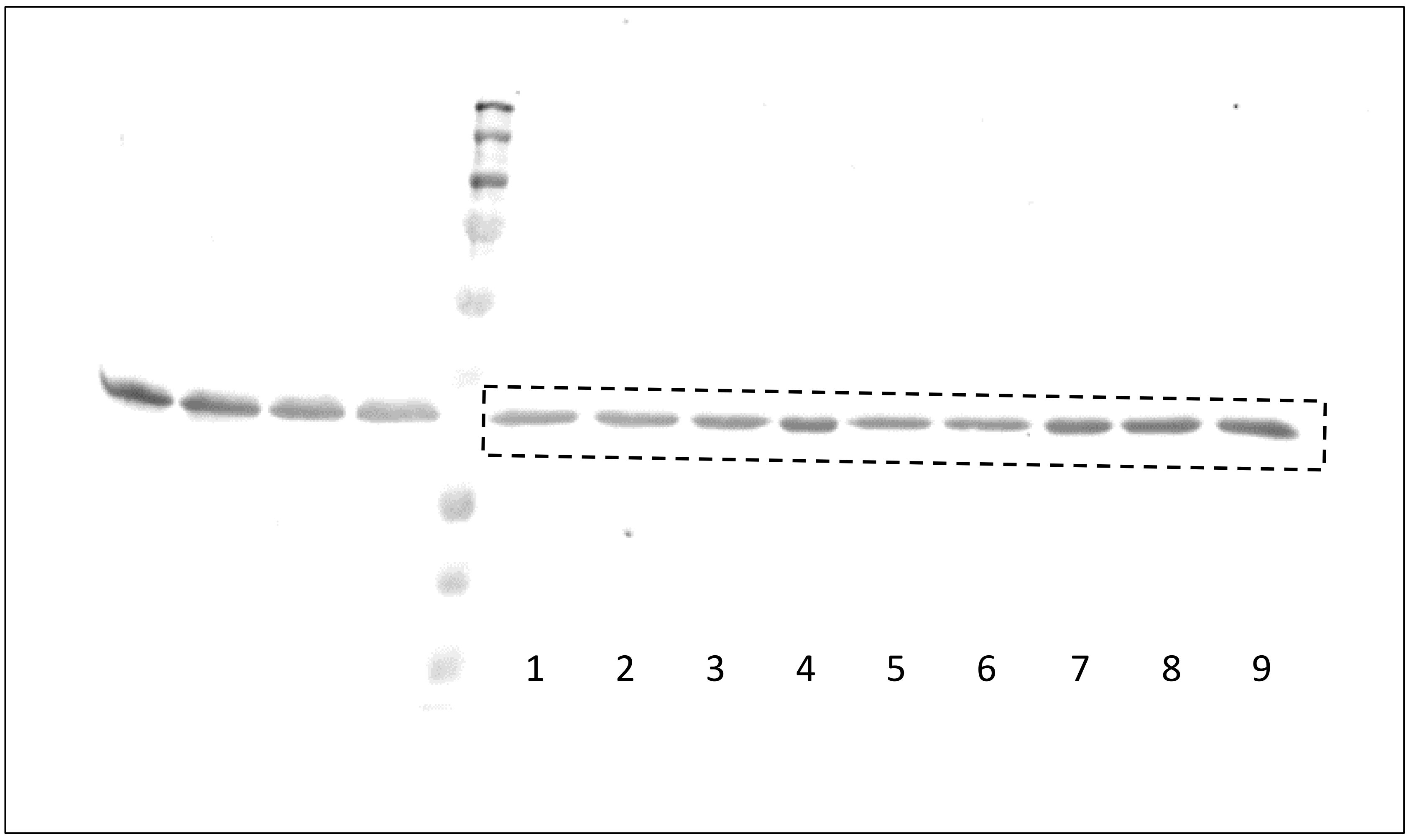

Anti GAPDH

Figure 8

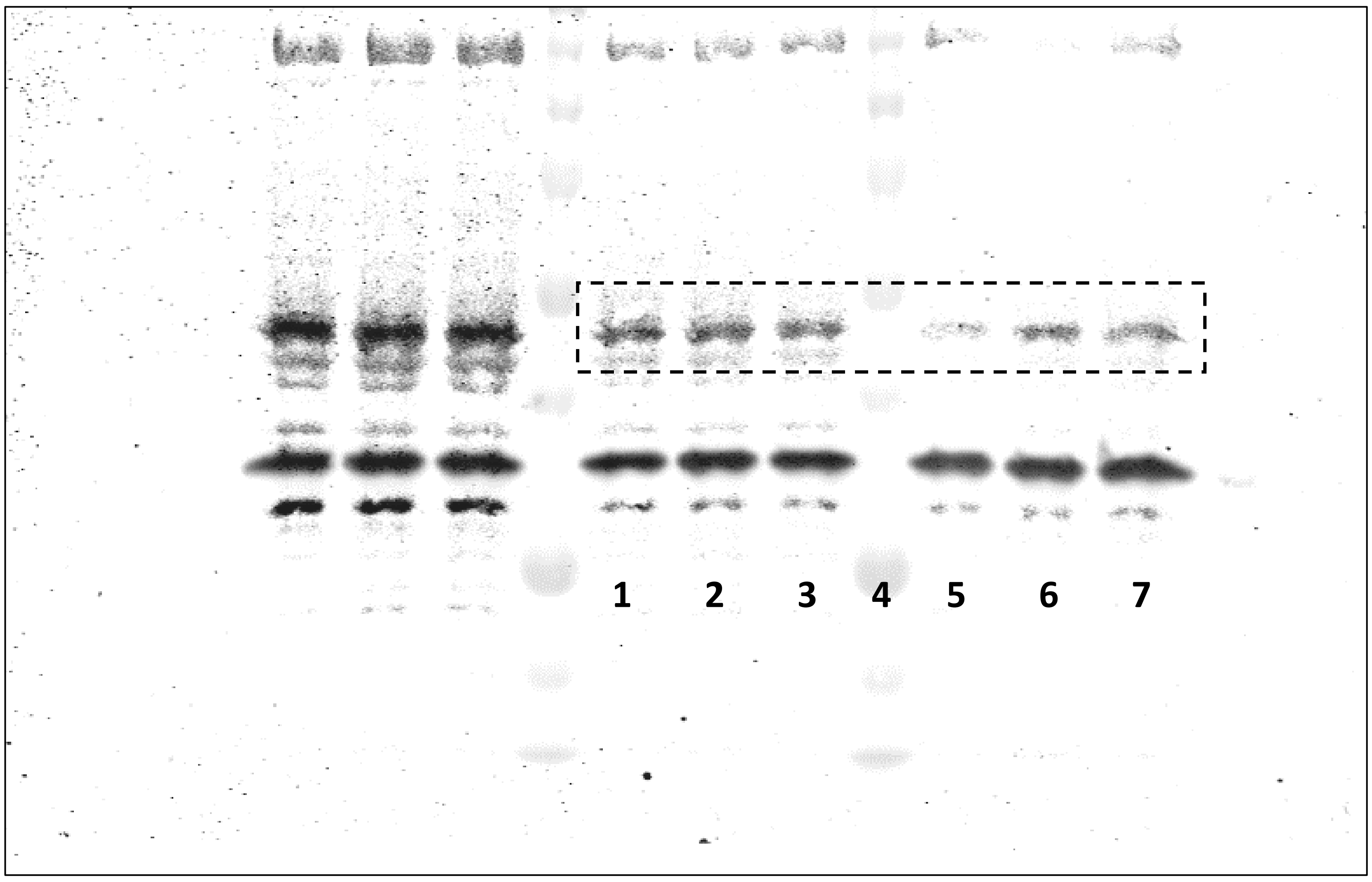

Anti PAH

1-3: WT liver
4: marker
5-7: : DNAJC12 KO liver

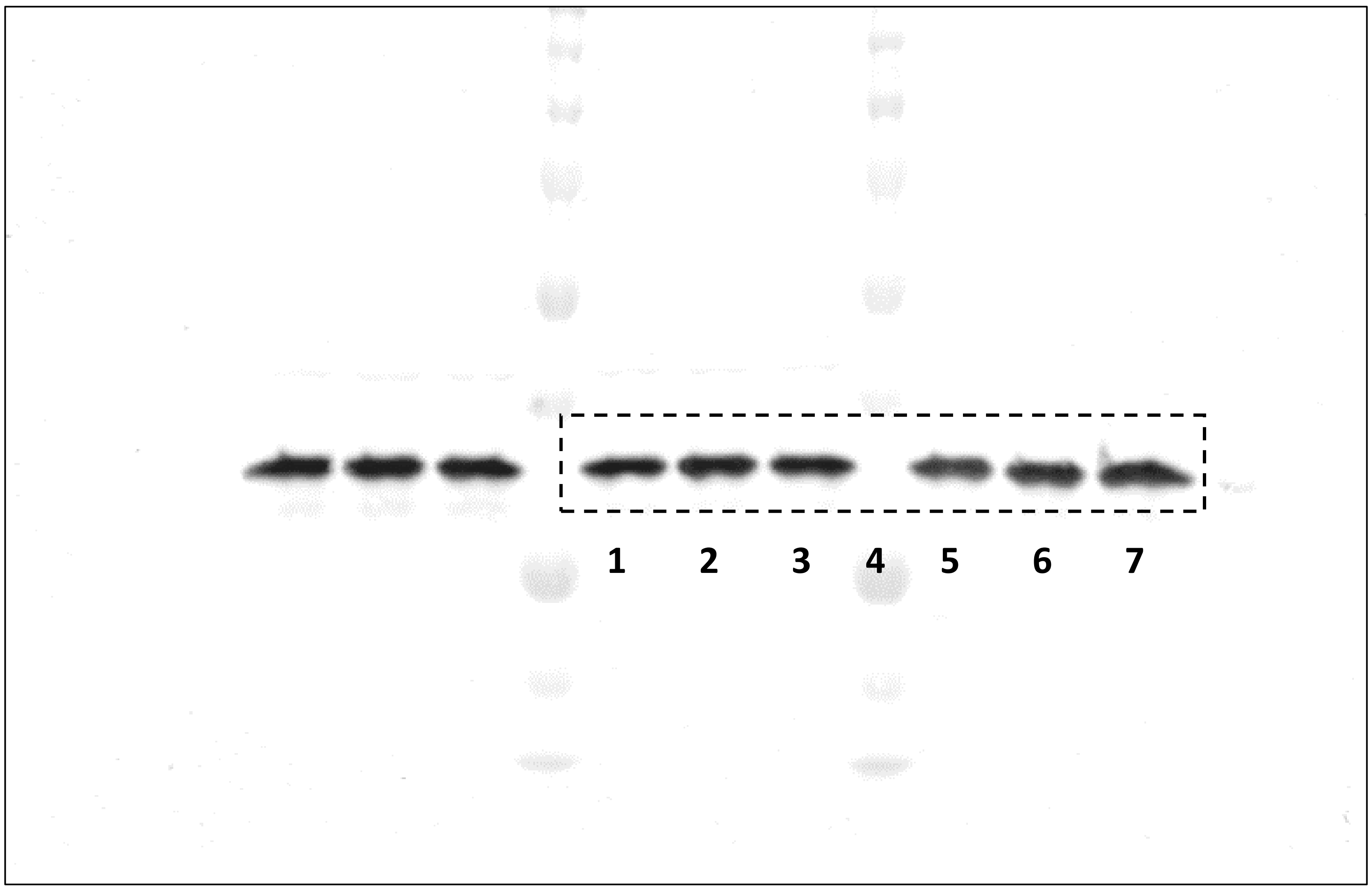

Anti GAPDH
