# Supplementary material for: Hyperphenylalaninemia and serotonin deficiency in Dnajc12-deficient mice
Source: Commun Biol. 2024 Dec 18;7:1641. doi: 10.1038/s42003-024-07360-6 (PMC11655531; doi:10.1038/s42003-024-07360-6)
Supplement: Supplementary file 2 — Description of Additional Supplementary File [file 42003_2024_7360_MOESM2_ESM.pdf]

Description of additional supplementary file

File name: Supplementary Data 1

Description: The source data behind the graphs in the paper
